# Unveiling GruPol: Predicting Electric and Electrostatic Properties of Macromolecules via the Building Block Approach

**DOI:** 10.1021/acs.jpcb.4c03062

**Published:** 2024-07-08

**Authors:** Raphael F. Ligorio, Paul Grosskopf, Leonardo H. R. Dos Santos, Anna Krawczuk

**Affiliations:** † Institute of Inorganic Chemistry, University of Goettingen, Tammannstrasse 4, D-37077 Göttingen, Germany; ‡ Departamento de Química, Instituto de Ciências Exatas, Universidade Federal de Minas Gerais, Av. Antônio Carlos 6627, 31270-901 Belo Horizonte, MG, Brazil

## Abstract

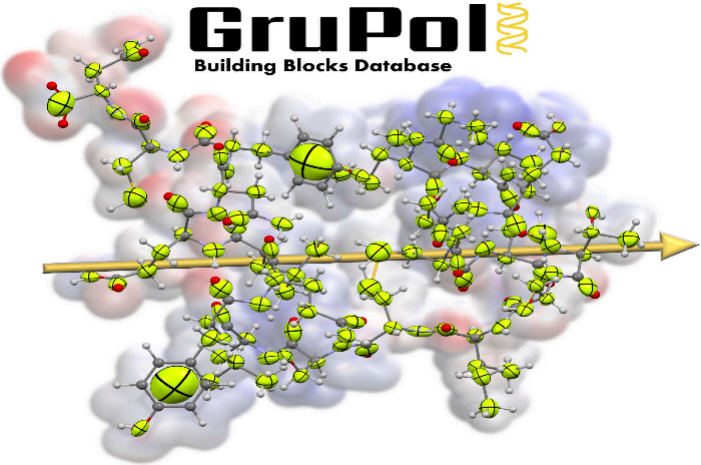

Understanding electrostatics and electric properties of macromolecules is crucial in uncovering the intricacies of their behavior and functionality. The precise knowledge of these properties enhances our ability to manipulate and engineer macromolecules for diverse applications, spanning from drug design to materials science. Having that in mind, we present here the GruPol database approach to characterize and accurately predict dipole moments, static polarizabilities, and electrostatic potential of proteins and their subunits. The method involves partitioning of the electron density, calculated at the M06-HF/aug-cc-pVDZ level of theory, of small peptides into predefined building blocks that are averaged over the database. By manipulating and positioning these building blocks, GruPol enables the description of proteins assembled from over nearly 100 residual entries, allowing for efficient and precise computation of the above-mentioned properties across a broad range of proteins. The database enables the user to include solvent effects as well as define protonation states on the protein’s backbone to account for pH variations. The precision of the proposed scheme is benchmarked against experimental data for myoglobin species.

## Introduction

Electrostatic properties involve interactions among stationary electric charges, encompassing phenomena like dipole moments and electrostatic potential. When considering how a molecule or material responds to an external electric field, it covers a range of electric properties, including its ability to polarize charges among different atoms or functional groups (polarizability), its response to the externally applied field (susceptibility), its capacity to store electrical energy (dielectric constant), and its ability to refract light (refractive index). Exploring those properties of proteins is indispensable for gaining profound insights into their structure, function, and interactions within biological systems. The distribution of charges within a macromolecule influences its overall electrostatic interactions, affecting its stability and reactivity. Dipole moments, arising from the separation of positive and negative charges, determine the molecule’s polarity, influencing its solubility and intermolecular forces. Polarizabilities reflect a macromolecule’s ability to respond to an external electric field, influencing its conformational flexibility and interactions with surrounding molecules. These properties collectively make possible to fine-tune desired functions, stability, or particular interactions. That facilitates the rational design of protein variants with customized electric properties, holding significant potential in the advancement of novel biocatalysts, therapeutic proteins, and materials across a spectrum of applications, including drug delivery systems and biosensors.^1−4^

In macromolecular research, accurately determining electron-density-related properties through *ab initio* quantum calculations is challenging due to the substantial computational requirements involved. However, one promising approach to address this challenge is to exploit the transferability of electron density observed across analogous systems. Using the transferability of chosen parameters, one can interpret large macromolecular structures in terms of smaller building blocks, such as atoms or functional groups. This approach bypasses the need for computationally demanding calculations on the entire macromolecule, enabling more efficient and targeted analyses of electron density phenomena in complex molecular systems. As an illustration of such conduct, one can cite here numerous databases—MATTS (former UBDB),^5^ ELMAM2,^6^ and GID (former Invariom)^7^—that utilize the transferability of multipole parameters to better describe electron density distribution in various systems including macromolecules. All of them highly concentrated on accurately approximating the electrostatic properties of large-scale systems, such as proteins; however, none of them is yet able to explicitly incorporate polarization effects of molecular electron density caused by interactions with neighboring molecules. Some attempts to correct for such effects were recently published;^8^ however, polarization effects were only partially included, and no effects coming from chemical bonding were discussed, thus neglecting the delocalization of the electron density toward regions between atoms. Nearly simultaneously Ernst et al.^9^ proposed a dedicated database to store information on polarizabilities, however focusing on functional groups rather than atomic contributions. Polarization effects experienced by highly polarizable atoms are too sensitive to subtle changes of the chemical environment; thus, the transferability concept may not be effective, leading to inaccurate property prediction. Instead, bigger building blocks such as functional groups have demonstrated efficacy in resolving this matter, and numerous advances on expanding that idea, particularly for biological systems, have been published.^10−13^

In order to harvest functional groups properties from molecules, hence enabling the establishment of polarizability database, a partitioning scheme must be used to cut the electron density into atomic fragments. Despite many available methods,^14−18^ including the Hirshfeld approach or the wave function partitioning scheme proposed by Stone,^19^ special attention is given to the hard-space partitioning introduced by Bader, namely Quantum Theory of Atoms in Molecules (QTAIM).^20^ QTAIM provides unique atomic boundaries and atomic connectivity and thus allows to exactly decompose any molecular electronic property into atomic contributions. Moreover, QTAIM is particularly useful for our purposes since it provides unique atomic dipole moments, which are independent of the origin,^21^ hence allowing exportability of atoms and functional groups.^9,22^ While a reasonable estimation of these properties can be derived from gas-phase calculations, their true attributes emerge within the intricate chemical environment of biomolecules. A comprehensive understanding of their behavior requires meticulous consideration of intermolecular interactions, as for example in the case of solvent–protein assemblies. Because of the relatively weaker nature of these interactions compared to covalent bonds, researchers can rely on methodologies based on classical electrostatics, consequently drawing insights from gas-phase properties, serving as a bridge to understand their behavior in condensed phases.^22−26^ In such approaches, the electric field experienced by a molecule in a condensed phase arises from the summation of an externally applied field and the contributions from adjacent dipoles affecting the initial gas-phase electric properties, as the case of those used in our software GruPol. In this context, given the critical role of aqueous environments in biochemistry, GruPol corrects the electric properties of a protein embedded by water molecules via a dipole interaction model.^27^

**Figure 1:**
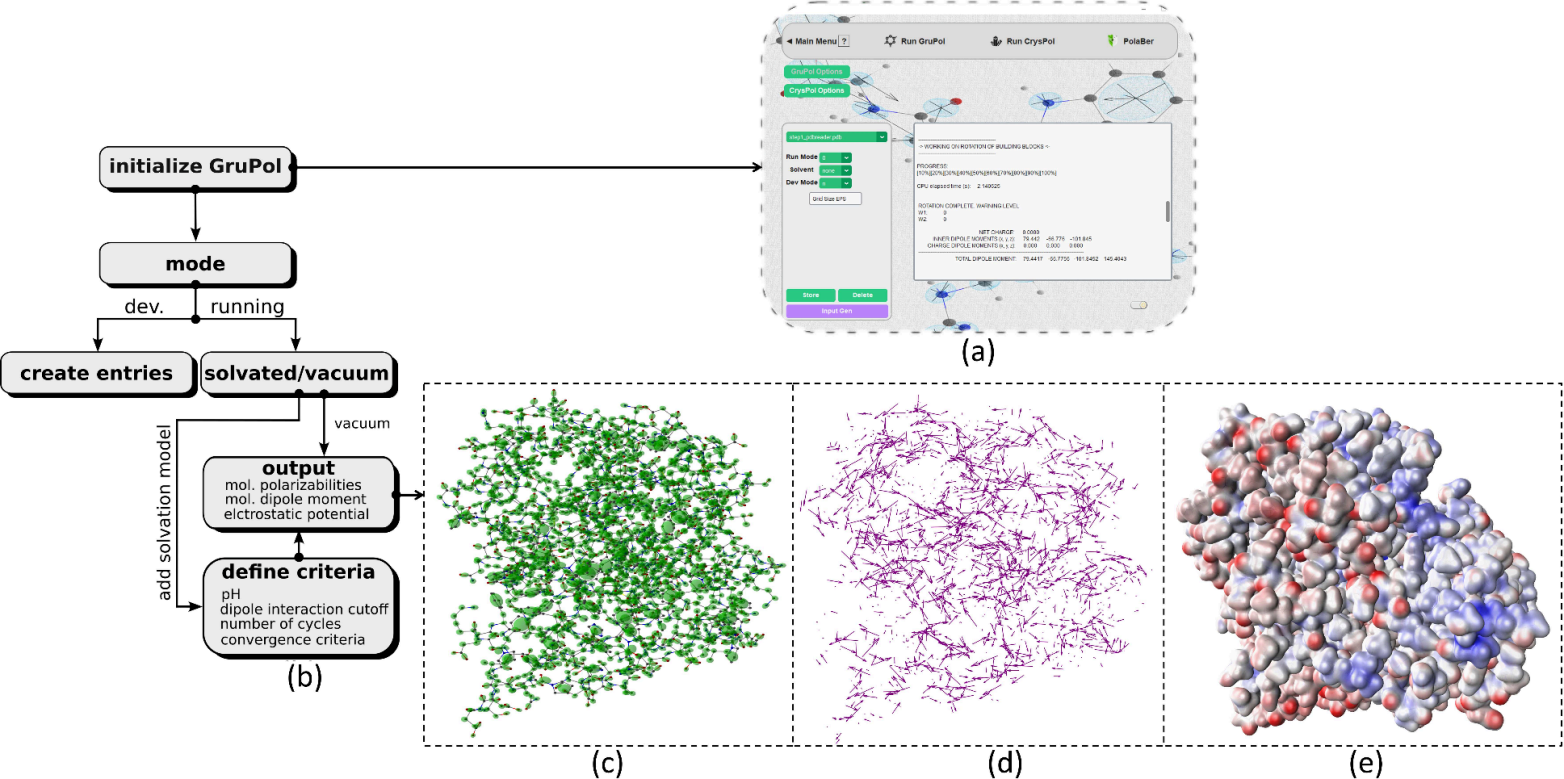
Overview of GruPol: (a) user interface, (b) schematic representation of user options, (c) graphical representation of group polarizabilities, (d) group dipole moments, and (e) GruPol-predicted electrostatic potential of Human Aldose Reductase (PDB code: 2R24). Group polarizability ellipsoids are drawn with a scaling factor of 0.2 Å^–2^, while group dipole moment vectors are represented at twice their original magnitude. Electrostatic potential is mapped over an iso-promolecular density surface at 0.01 au. Values range from −0.35 au (red) to +0.35 au (blue).

A fundamental aspect of our work involves the establishment of a comprehensive software, namely GruPol, containing a database encompassing building blocks dipole moments and static polarizabilities for the most common 20 amino acid residues. With the current version, we emphasize its proficiency in gauging molecular and functional group properties by assembling a designated protein structure using distinctive building blocks. Our database excels in predicting dipole moments and polarizabilities, demonstrating its versatility across both gaseous and solvated phases, while offering the flexibility to simulate the pH of the solution to specific requirements. Notably, a key aspect of our program lies in its facilitation of electrostatic potential (ESP) estimation through the strategic utilization of group dipole moments.^12^ In a nutshell, GruPol stands as an innovative approach allowing the precise predictions of electric and electrostatic properties of macromolecules. The overview of GruPol’s capabilities is shown in Figure 1.

## Theoretical Methods

### Quantum Mechanical *Ab Initio* Calculations

Electron density determination was performed at the M06-HF/aug-cc-pVDZ level of theory using Gaussian 16 software.^28^ This choice was motivated by the efficiency of meta-hybrid functionals, particularly the M06 family, in accurately reproducing polarizabilities and dipole moments when compared to reference methods like coupled cluster with single and double excitations.^22,29−31^ The electron density was partitioned according to Bader’s Theory of Atoms in Molecules (QTAIM) using AIMAll software.^32^ For atomic properties, we utilized PolaBer^33^ to compute origin-independent dipole moments and distributed polarizabilities. Database entries, i.e., dipole moments and polarizabilities of functional groups (see the Supporting Information, Section S8), were extracted from a set of 28 peptides in their respective neutral zwitterionic states (about 5 residues each), generated in a randomized manner and subsequently optimized using the CHARMM^34^ force field within a gas phase environment. It is important to note that while the properties stored within the database remain unaffected by the origin of the building block, they do rely on the orientation of each functional group in relation to the Cartesian axes. For this reason, a subsequent rotation of the building block to a common framework was further performed based on the charge tensor of the functional group, analogous to the inertia tensor, in which masses are replaced by atomic numbers.^9^ A detailed discussion of how the GruPol entries are obtained is given in the Supporting Information, Section S3. Notably, all peptides were maintained in the terminal neutral zwitterionic configuration throughout the analysis. Furthermore, a diverse variety of nearly 100 building blocks were obtained (see Table S1), and a given building block entry was collected from a minimum of five distinct molecules, ensuring a varied chemical environment while preserving similarities across the data set. The mean values for each specific building block property were calculated and used throughout.

To validate GruPol, peptides with up to 100 residues were employed as a testing set. The set was subject to the same conditions as those used for generating entries in the database: (i) terminal neutral zwitterion state and (ii) geometries optimized in the gas phase using the CHARMM force field. Because of the challenges in achieving SCF convergence when using the M06-HF/aug-cc-pVDZ level of theory for large molecules, the validation of the database entries was performed using two distinct testing subgroups. The first one comprised 13 molecules ranging in size from 10 to 100 amino acid residues. In this subgroup, molecular polarizabilities and dipole moments were estimated using the M06-HF/cc-pVDZ method. The second subgroup consisted of 11 molecules ranging from 8 to 12 residues, for which the electric moments were calculated using both M06-HF/cc-pVDZ and its diffuse counterpart, aug-cc-pVDZ, being the latter employed for creating GruPol’s entries. The comparison between the two basis sets confirmed that the effect on dipole moment estimation is overall negligible (see Supporting Information Section S7). For this reason, GruPol provided reliable results for both subgroups. However, the inclusion of augmented functions has a significant impact on achieving accurate polarizabilities, as the nondiffuse basis set tends to underestimate both atomic and molecular quantities.^29^ The overall differences in the main diagonal components between the two basis sets is approximately 20%, whereas off-diagonal components show similar behavior regardless of the basis set employed.

### Atomic Properties

Atomic dipole moments obtained via QTAIM can be defined as a summation of the two following components:
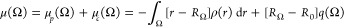
1where *q*(Ω) is the atomic basin charge, *R*_Ω_ is the vector position of Ω, and *R*_0_ is the origin of an arbitrary coordinate system. The polarization component μ_*p*_(Ω) is obtained by integrating over the atomic volume, hence not origin dependent. The charge-translation term μ_*c*_(Ω) can be rewritten as an origin-independent contribution that allows for the transferability of properties from one molecular system to another:^21^
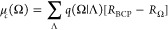
2where *q*(Ω|Λ) represents the charge induced on the atomic basin Ω due to its bond with another atomic basin Λ. The position vector *R*_BCP_ corresponds to the bond critical point between Ω and Λ, measured with respect to the arbitrary origin *R*_0_.

Once origin-independent atomic dipole moments are at hand, the polarizability tensor components of a given atomic basin are calculated by numerically differentiating the dipole moment with respect to the applied electric field along the ±*X*, ±*Y*, and ±*Z* axes. This procedure is exact provided that the perturbation of the field is small enough to guarantee linear response, e.g., 0.005 au or lower. The α_*ij*_(Ω) component of the tensor is given by
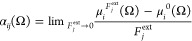
3The atomic polarizability tensors can be visualized in the same space as the molecule, assuming 1 Å^3^ ≡ 1 Å. The isotropic polarizability is obtained by averaging the main diagonal components, while the anisotropy of the tensor is estimated using eq 4:
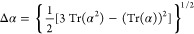
4For further details, the reader is referred to a paper by Krawczuk et al.^33^ It is worth noting here that PolaBer was used only to generate the database entries, being GruPol an independent software and its utilization does not rely on external programs.

Following calculations and extraction of functional groups, dipole moment vectors and polarizability tensors are aligned based on a predefined framework determined by the geometry of each functional group. Because of their orientation dependence, the initial step to create an exportable building block involves aligning equivalent groups to a standardized framework. This alignment is accomplished using a rotation matrix derived from the diagonalization of the charge tensor (*Q*), analogous to the inertia tensor, where atomic masses are substituted with atomic numbers (*Z*).
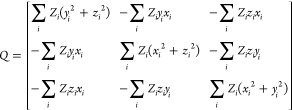
5where *x*_*i*_, *y*_*i*_, *z*_*i*_ are the atomic coordinates of a given building block referred to an arbitrary coordinate system in which the origin is the center of charges of the respective building block.

The charge-density distribution of a building block, along with its associated dipole moment and polarizability, is influenced by the surrounding charge distribution. To illustrate, consider the properties of a −CH_2_– group extracted from two different molecules: methane (highly symmetrical) and glycine (less symmetrical). In the case of methane, the charge density of the −CH_2_– group retains the same symmetry as the idealized −CH_2_–, which is *C*_2*v*_. However, in the case of glycine, the presence of different atoms bonded to the carbon reduces the effective symmetry of the building block to *C*_*s*_. In the context of a *C*_2*v*_ charge density distribution, after rotation, all off-diagonal terms in the polarizability tensor are expected to be zero. Conversely, for the *C*_*s*_ symmetry, the off-diagonal components are not necessarily nullified. To ensure consistent components after group rotation, neighboring atoms are explicitly included in the computation of the transferable functional group. This implies that our −CH_2_– building block is actually represented as a R_1_–CH_2_–R_2_ group, where R_1_ and R_2_ serve as placeholders or dummy atoms. The definition of each building block is given in Supporting Information Section S3.

### Solvent Inclusion and Molecular Dynamics Simulation

Given the biochemical aspects of the database, it seemed natural to include solvent effects within GruPol and benchmark the results against experimental values, i.e., dipole moments. To showcase the performance of the database, we have chosen two myoglobin proteins: HH myoglobin extracted from horse heart (PDB code: 1WLA) and SW myoglobin identified in sperm whales (PDB code: 1MBN). Water–protein aggregates for both SW and HH myoglobins were built by employing CHARMM-GUI^35^ feature (cubic box with sides of 180 au). Water–protein cluster geometries were optimized using the Adopted Basis Newton–Raphson Method, utilizing CHARMM additive force field version 46b1.^34^ Protein’s backbone was chosen in such a way to ensure its neutral-terminal zwitterion state as well as neutral solvent molecules. Subsequently, an equilibration step was performed in the NVT ensemble, comprising 100000 steps with a time interval of 1 fs. Temperature control was achieved using the Nosé–Hoover^36^ thermostat set to 303.15 K. Following this, molecular dynamics simulations were performed in the NPT ensemble, controlling both temperature and pressure at 303.15 K and 1 atm, respectively.^37^ The simulation was carried out for a total of 200000 molecular frames with a time interval of 2 fs, corresponding to an overall simulation time of 400 ps. Geometries were extracted every 16 ps.

To estimate the impact of solvent effects on the dipole moments of the studied proteins, a dipole interaction model was used.^27^ This model employs gas-phase derived dipole moments and polarizabilities, here GruPol’s building blocks quantities, to incorporate changes in these properties induced by the chemical environment, in our case solvent molecules. Equation 6 clarifies how condensed phase dipole moments can be computed from their original gas phase equivalents:
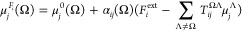
6where *F*_*i*_^ext^ is the external electric field applied in the direction *i* and the distance tensor *T*_*ij*_^ΩΛ^ is defined as follows:
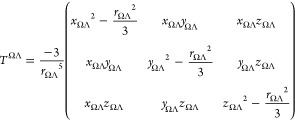
7where *x*_ΩΛ_ = (*x*_Ω_ – *x*_Λ_) is the difference in the Cartesian *x* coordinate between the basins Ω and Λ (respectively for *y* and *z* directions) and *r*_Ω_ Λ is the associated distance between two building blocks located at their respective center of charges (atomic numbers).

Considering the solvent’s capacity to induce local charges, the precise determination of electrical properties of a given peptide is only reached accounting for the protonation of titratable residues. Different values of pH lead to different charge distributions on the protein’s backbone, depending on the p*K*_a_ of each residue.^38^ These differences affect primarily the overall molecular dipole moments as well as the building blocks ones.^12^ Since a given charge distribution produces an internal electric field, modifications on dipole moments can be done replacing the term between the parentheses in eq 6 with the internal electric field, thus obtaining corrections on the so-called core dipole moment. Subsequently, a dipole interaction model (DIM) can be applied using the new core dipole moments. Additionally, corrections due to the charge separation must be taken into account, termed the charge dipole moment. Section S6 in the Supporting Information provides a comprehensive description of how these effects were incorporated in the GruPol database.

## Results and Discussion

### Database Validation

When comparing GruPol’s molecular polarizabilities with those obtained via *ab initio* quantum calculations, the overall difference for the main diagonal components is approximately 5% when using a diffuse basis set and 15% when using its non augmented offspring. This highlights GruPol’s ability to potentially offer more precise polarizability values compared to *ab initio* quantum calculations in the absence of diffuse functions. Polarizability’s diagonal components rely more on the covalent bonds as the principal axes of the ellipsoids are aligned with them. Consequently, the off-diagonal terms are more influenced by intermolecular interactions, which have a lesser impact on the orientation of the ellipsoids. Hence, the accuracy of the off-diagonal components is lower, primarily because they are more dependent on the chemical environment rather than intramolecular bond formation. Nevertheless, these components correspond to less than 10% of all diagonal components, thus not affecting the overall good agreement between GruPol and *ab initio* quantum calculations.

**Figure 2:**
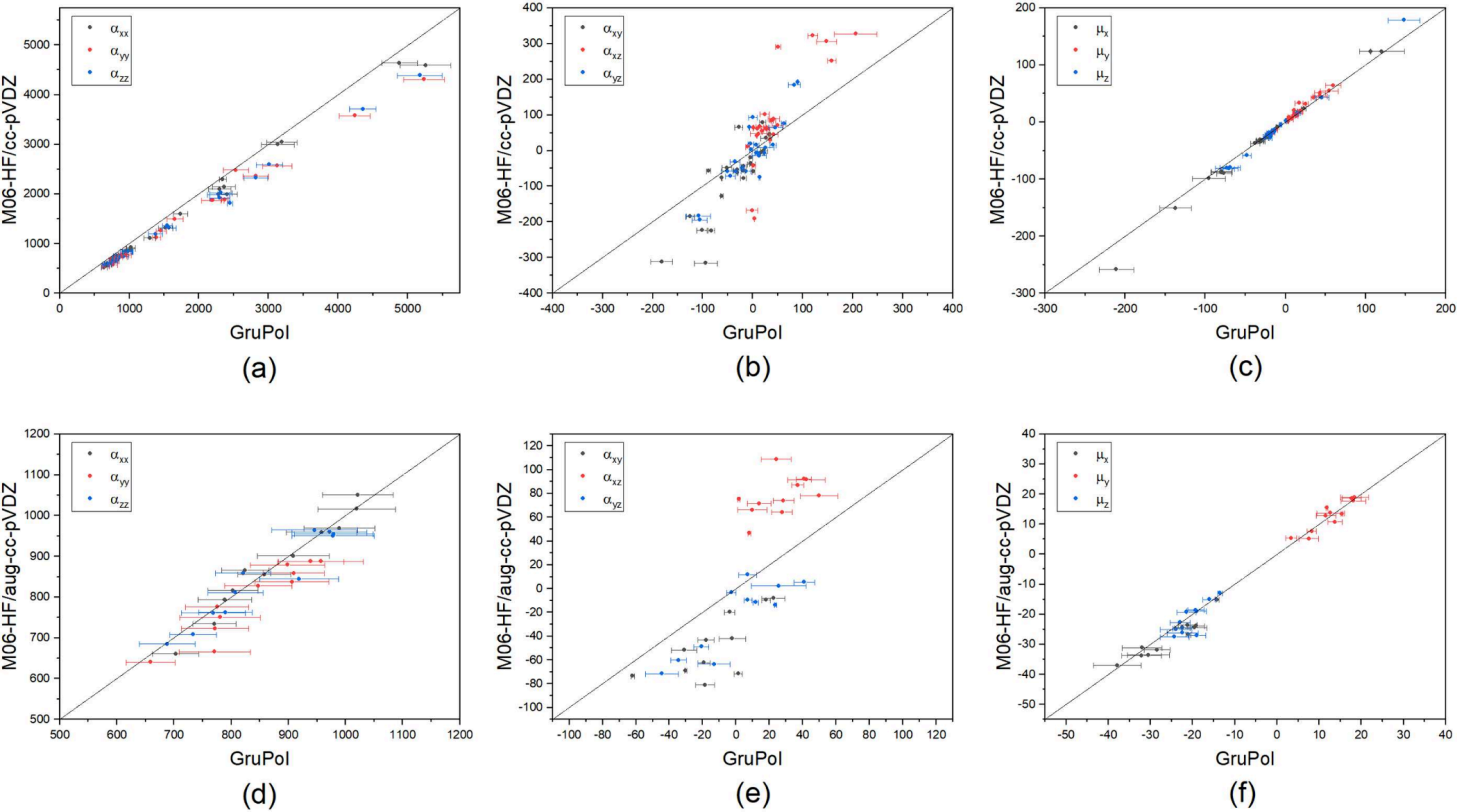
Database comparison with *ab initio* quantum calculations at the M06-HF/cc-pVDZ level of theory for the main diagonal components of molecular polarizability (a), off-diagonal components (b), and molecular dipole moments (c) for both subgroups. Same quantities are respectively depicted (d, e, f) for the second subgroup of the benchmark peptides, encompassing peptides from 8 to 12 residues, calculated at M06-HF/aug-cc-pVDZ. Overestimation of molecular polarizabilities of about 15–20% when comparing GruPol with quantum calculations employing a nondiffuse basis set is expected, given that the database was built employing diffuse functions. Error bars represent the summation of the confidence interval (95%) of each individual entry.

In terms of dipole moments, the overall offset is approximately 10% for the magnitude and 5° for the angle, indicating that the GruPol database accurately describes the size and what is equally crucial, the direction of the dipole vector. A comparison between GruPol and *ab initio* quantum calculations is shown in Figure 2. Detailed results and description concerning the chosen molecules for benchmarking studies can be found in Supporting Information Section S7.

### Electrostatic Potential Maps

The estimation of electrostatic potential (ESP) in large molecules is commonly performed using charge distribution methods.^39^ Nevertheless, that is not the only possible route. Our recent studies^12^ have shown that for small peptides, instead of atomic charges, one can use atomic dipole moments and anyway accurately reproduce the ESP calculated through quantum methods, particularly in regions close to the limits of the van der Waals radii. From the distributed atomic moments, the electrostatic potential due to the presence of a given basin is defined as
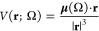
8where **r** is a position vector at any point in space. Given the additivity of QTAIM-derived parameters, summation of *V*(**r**;Ω) over all atomic basins provides the molecular electrostatic potential mapping. Atomic contributions can be further redefined into the functional group dipole moment-based electrostatic potential (GEP), which is a simplified version of the atomic-based ones (AEP). The advantage of such an approach lies in a straightforward definition of a building block that is at the foundation of the GruPol database. A detailed discussion on the methodology can be found in the Supporting Information (Section S5).

**Figure 3:**
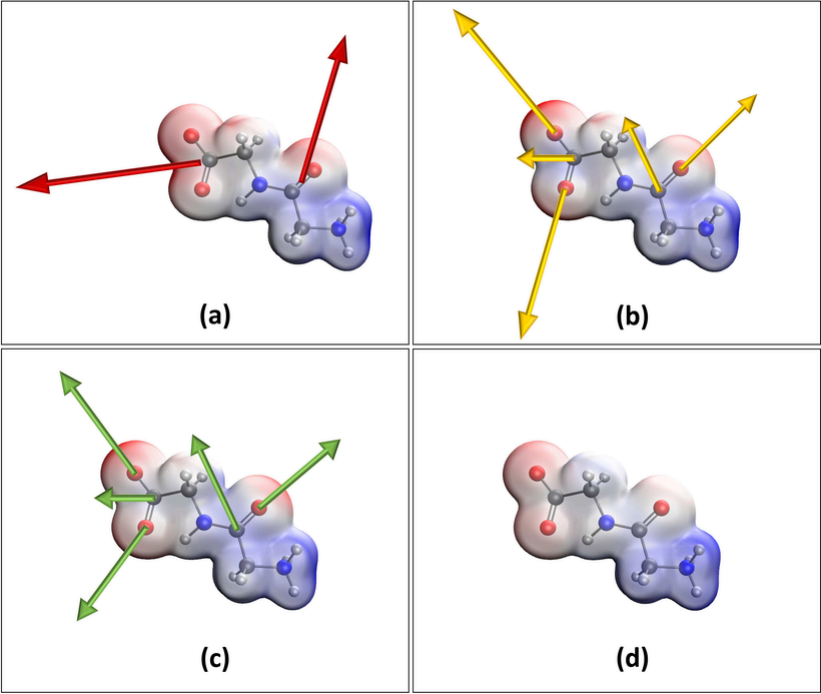
Electrostatic potential of the Gly–Gly dipeptide calculated with (a) group dipole moments approach, (b) GruPol using the split model, (c) atomic dipole moments, and (d) *ab initio* (M06-HF/aug-cc-pVDZ), plotted over an isodensity surface at 0.01 au obtained at the same level of theory. Values range from −0.35 au (red) to +0.35 au (blue). The dipole moment vector is plotted following the convention, from the positive to the negative charge.

Although our first attempts^10,11^ to reproduce ESP of polypeptide chains, employing group dipole moments, gave very promising results, a closer look at some functional groups, e.g., carboxylic groups or peptide bonds, indicated an incorrect distribution of the electrostatic potential. In particular, one could expect that the most electronegative ESP shall be located on the oxygen atoms in the region of lone pairs. Instead, we observed the electronegative area being located exactly between the two oxygen atoms (see Figure 3a). Such a behavior is a consequence of the way how a group dipole moment is defined.

By averaging the atomic contributions into a group property, we sum up the vectors of individual atoms, and the resulting orientation of the group dipole moment neglects the natural behavior of individual atoms in a functional group. For most of the functional groups, this artifact is negligible; however, for those where anisotropy is a natural property, atomic effects should not be overlooked. To address this issue, we adopted a strategy that considers the atomic distribution and corrects the electrostatic potential reflecting its correct distribution. A general solution consists of splitting the dipole moments of NH(CO) and COO^–^ groups in such way to mimic the atomic contributions used to form the group dipole moment. For further details on the decomposition procedure, the reader is referred to Section S5 in the Supporting Information. It is important to mention that using group dipole moments instead of atomic ones decreases the accuracy of the predicted ESP of all groups. However, for most building blocks, especially those formed by H atoms and a unique non-H atom (including here C, N, O, and S ones), the decrease in accuracy is not very noticeable. This is because the group’s dipole moment mainly comes from the non-H atom, as is the case with the −NH_3_^+^ group.^40^ It is also worth mentioning here that the atomic electric properties are in many cases too sensitive to the subtle changes of the environment; thus, transferability is not accurately achieved as in the case of functional groups.^9^ That justifies the fact that the database was built employing building blocks properties rather than atomic ones. Therefore, we opted for the methodology of splitting the building block dipole instead of creating new atomic entries for specific groups. As Figure 3b shows, the splitting methodology precisely reproduces the electrostatic potential calculated through the AEP approach (Figure 3c). Nonetheless, one must be aware of the fact that this mechanism may introduce some additional imprecisions to the database, and the resultant electrostatic potential may be less accurate than the one obtained directly from atomic contributions. For this reason, we are currently assessing the accuracy of storing the atomic parameters, thus suppressing the necessity of using the splitting methodology, at least for some of the functional groups, where we observe the most of the discrepancies in the distribution of electrostatic potential. Also, the purpose of the current studies is not to focus on the precision of atomic ESP (AEP) compared to *ab initio* ESP, as this has been addressed in prior research,^12^ but rather to demonstrate that GruPol yields analogous outcomes to AEP, albeit utilizing group dipole moments instead of atomic ones.

**Figure 4:**
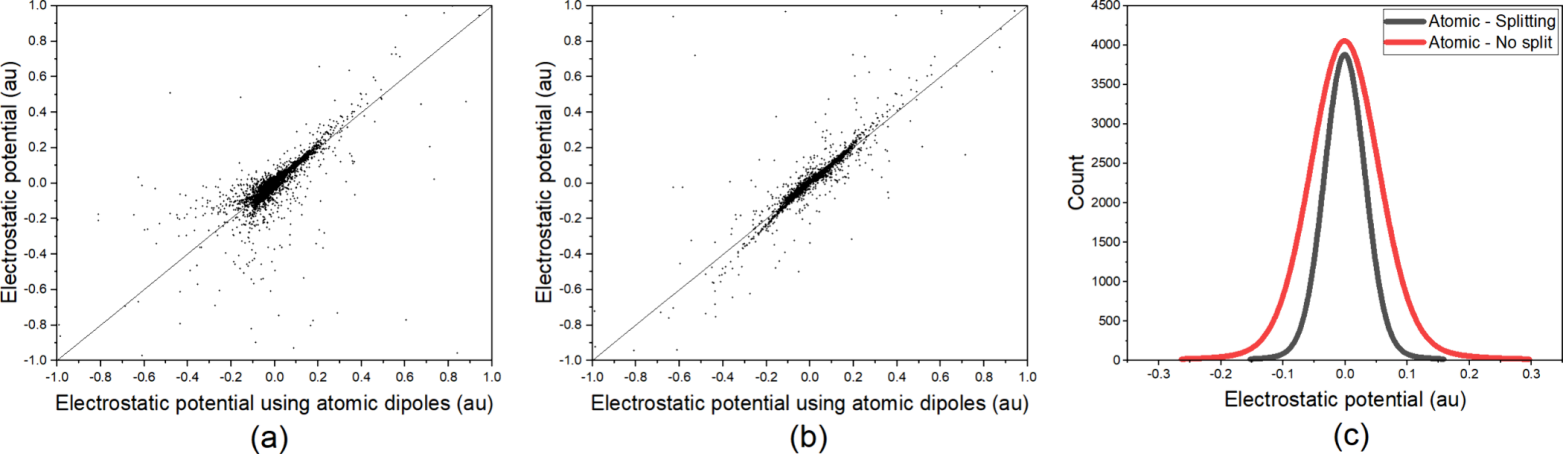
Comparison of the Gly–Gly electrostatic potential obtained from atomic dipole moments (AEP) with the ESP calculated for every point in the grid surrounding the peptide using (a) a nonsplit model of group dipole moment (GEP) and (b) a split model and (c) differences between ESPs calculated applying both models and the reference.

As a proof of concept, we once again used the Gly–Gly dipeptide prototype. In Figure 4 a graphical representation is given, comparing both approaches, GEP and splitting dipoles, with a more precise calculation, AEP, and showcasing the improvement in GruPol’s electrostatic potential predictions. The ESP was calculated employing the three different methods across a grid of 6555 points, with a 1 au spacing in each direction (23 × 15 × 19 points for *x*, *y*, and *z*). To determine the volume encompassing the peptide, atomic coordinates were sorted, and 5 au was added and subtracted from the maximum and minimum values along each Cartesian axis, respectively. Consequently, the middle point in each direction serves as the origin of the enclosing box. Since the peptide bond group is the most common building block in proteins, we anticipate that this enhancement would be even more necessary for larger proteins, given the increased number of peptide bonds. However, it is important to note that for macromolecule as the one present in Figure 1, comparing AEP and GEP is not possible due to computational limitations. The process of obtaining group dipole moments through GruPol’s method proves to be remarkably time efficient, requiring only a few seconds for the human aldose reductase (Figure 1e), serving as an example of macromolecules. Conversely, the duration of electrostatic potential computation is contingent upon the number of points in the grid surrounding the molecule. For this particular protein, and those built from similar number of residues, estimation of ESP is done within nearly a minute, when a grid spacing of 1 au is employed. These computations were executed on a single core, utilizing an Intel(R) Xeon(R) W-1290P CPU at 3.70 GHz.

### Water Medium Properties

Macromolecules in general present challenges for accurate determination of their electrical and electrostatic properties using theoretical approaches, as *ab initio* quantum calculations become impractical due to the size of these molecules. For this reason, explicitly adding solvent molecules in such calculations are nearly unfeasible. The exploitation of explicit solvents is more suitable for classical and semiclassical methods, e.g., force fields in molecular mechanics, where the number of molecules can be scaled up to thousands of atoms, while ensuring timely computation. On the other hand, implicit solvent models find extensive applications in computational chemistry for mimicking solvent effects, eliminating the need for the explicit representation of a large number of molecules. However, these models often employ an isotropic approximation for polarization effects, neglecting the directional dependence of electron density in response to external electric fields.^41,42^ As a result, such models may provide unreliable properties, particularly for systems where anisotropic polarization significantly influences the studied properties, such as electric moments.

**Figure 5:**
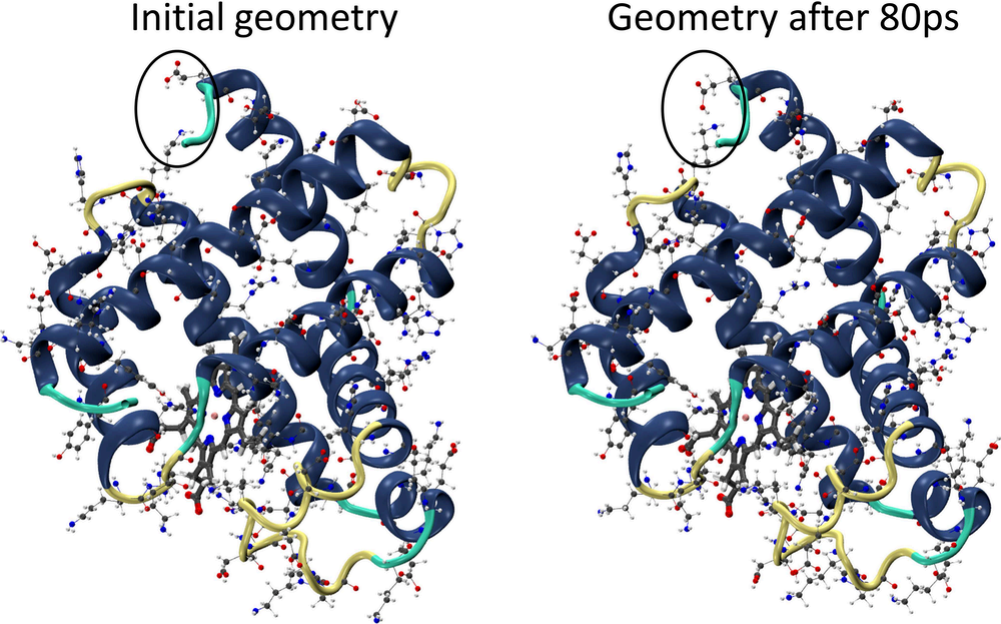
Secondary structure of SW myoglobin before (left) and after dynamic simulation (right). Titratable residues are represented with balls and sticks. The circled area indicates a formation of intramolecular interaction, capable of producing local charges.

To explore how solvent affects GruPol’s predicted properties, water molecules were explicitly introduced enclosing two proteins: SW and HH myoglobins. Subsequently, molecular dynamics simulations were conducted, hence grasping the inherent molecular flexibility of macromolecules in general. A dipole interaction model (DIM) was then used to assess changes in these properties, when shifting from gas to condensed phase, across a wide array of conformations. Differences in myoglobin species significantly influence the distribution of charged residues along their backbone, thereby affecting their electrical properties.^38,43−46^ Consequently, these variations impact folding, solubility, or reactivity.^46^ The influence on protein folding is directly observable in Figure 5, where titratable residues with opposite charges tend to approach each other, thus directly affecting the dipole moment. Naturally, the presence of local charges depends on the pH of the solution and the p*K*_a_ of each residue. For instance, drastic changes in pH can lead to protein denaturation, fundamentally changing its structure. Our simulations do not consider charged water molecules or charged residues apart from the terminal groups, thus neglecting different solutions’ acidity. Instead, we incorporate pH effects post-dynamic simulation, choosing pH values that ensure protein stability and integrity,^46,47^ thus preventing denaturation and maintaining proper comparability between GruPol’s predictions and experimental data. Nonetheless, it is important to recognize that this approximation could lead to errors when calculating dipole moments based on geometries obtained at different pH levels, and we anticipate it may be highly inaccurate during denaturation. Directly accounting for pH changes during the dynamics simulation would bypass this simplification; however, the current version of GruPol requires neutral residues to properly assemble and rotate the building blocks, meaning, for example, that acidic residues must be protonated. We are currently working on a modified code to identify building blocks according to their protonated state, thus eliminating the need for neutral species to recognize a given building block. This means that neither the definition of the building block nor its stored properties will change when a charge is added or removed; GruPol will automatically reassemble the building block to ensure proper rotation.

**Figure 6:**
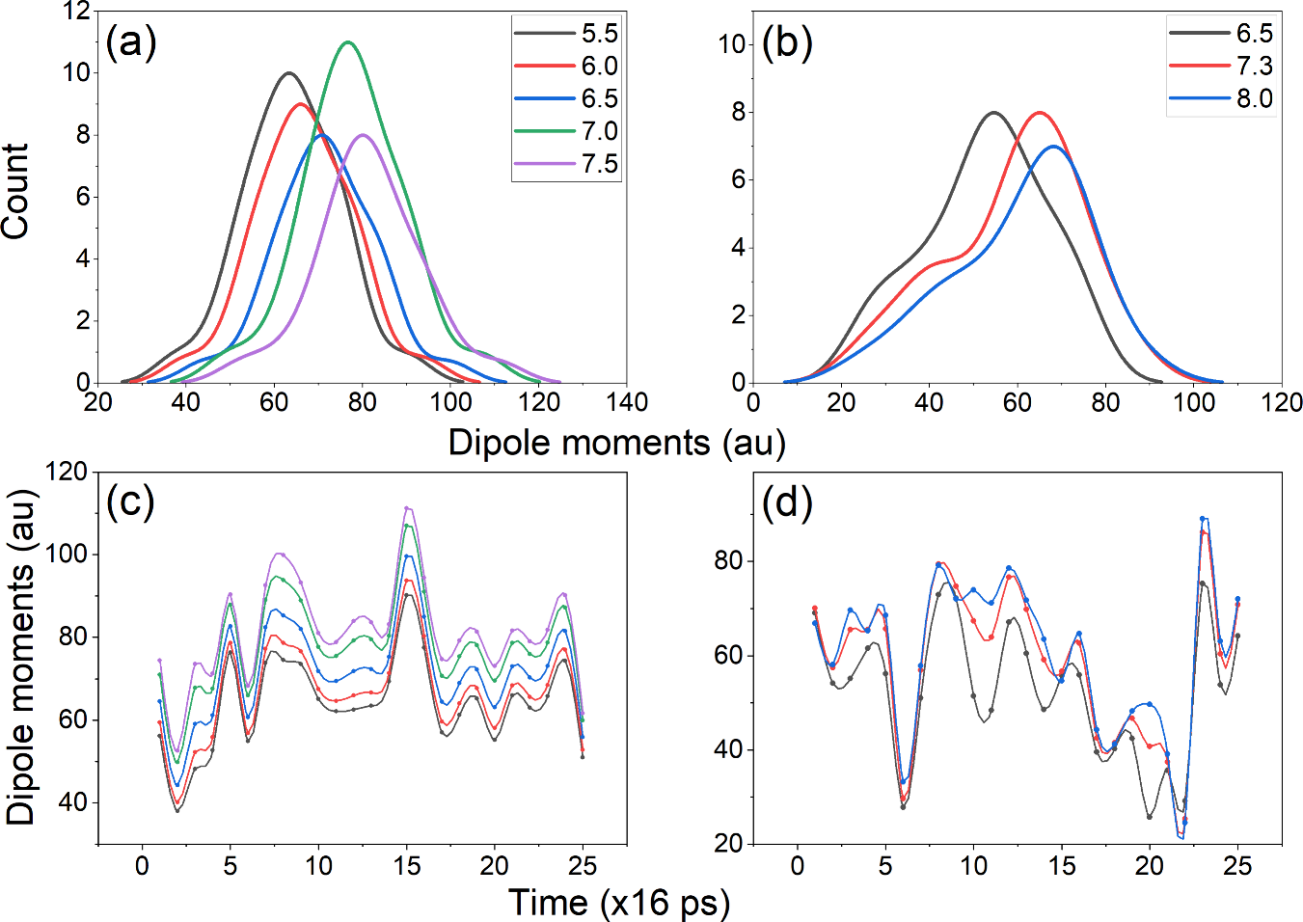
Distribution of dipole moments (absolute values) along molecular dynamics simulation for both HH (a) and SW (b) myoglobins. Time evolution of dipole moments for HH (c) and SW (d) myoglobins, calculated simulating different pH values. Dynamic simulations were performed with the zwitterion configuration, and charges on the protein backbone were further adjusted given a pH value, employing the GruPol approach.

Figure 6 illustrates the time-dependent behavior of the dipole moment for myoglobin at various pH levels. Notably, the dipole moment increases as the net charge on the backbone approaches neutrality for both myoglobin species. This can be potentially attributed to the isoelectric point, where an equilibrium exists between the total positive and negative charges, consequently increasing the charge dipole moment, influenced by the spatial arrangement of the titratable residues. The calculated isoelectric point for HH myoglobin species is found to be pH 7.3, while for SW species it is pH 7.5, consistent with existing literature reports.^45^ The maximum dipole moments correspond to these isoelectric points, indicating a dominant presence of charged groups. Supplementary analyses (see Supporting Information, Subsection S6.6) involving di-, tri-, and tetrapeptides of alanine oligomers demonstrate a symmetric dipole moment distribution centered around the isoelectric point. The curves in Figure 6c,d behave similarly over almost the entire range of pH, albeit with a shift toward higher dipole values when approximating the isoelectric point. However, certain conformers deviate from this pattern, particularly in the case of SW myoglobin. For example, frames around 160 and 320 ps exhibit a dip in dipole moment at pH 6.5 and 7.3, while displaying an increase at pH 8.0. Importantly, as the pH corrections were implemented after the dynamics simulation, the significant differences in the dipole moment curves in the above-mentioned regions do not imply an alteration in the protein structure. These variations can be rather attributed to the inherent charge displacements stemming from changes in the charge arrangement within the structure, affecting both the core and the charge dipole moments.

**Figure 7:**
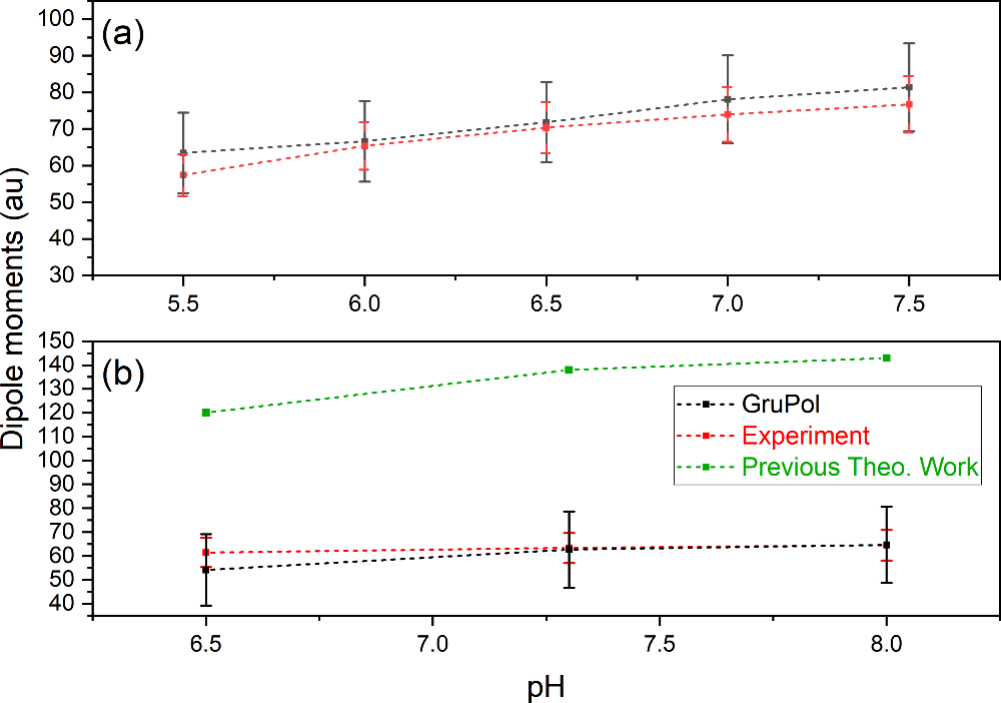
Experimental^47^ comparison of myoglobin dipole moments with GruPol results for HH (a) and SW (b) at various pH. Error bars associated with GruPol’s predictions were calculated using the standard deviation of the dipole moments of each conformer gathered during the molecular mechanics simulation. Previous theoretical work^38^ shows the same trend difference of experimental and GruPol’s prediction, but nearly two times overestimated. It is important to note that the previous theoretical work aimed at differences on p*K*_a_ shifts on the estimated dipole moments, and the results reported there significantly vary according to the set of p*K*_a_ used for the titratable residues.

Various theoretical approaches exist in the literature,^4,45^ primarily estimating molecular dipole moments based on charge distribution. However, they frequently result in over- or underestimations due to the absence of polarization corrections, from either the solvent molecules or the polarization effects rising from the internal charge distribution,^38^ particularly for those molecules where charged functional groups in the backbone are present. By combining dipoles and polarizabilities, GruPol offers reliable predictions of those electric moments for either charged, neutral, or zwitterionic forms of proteins. The agreement between GruPol results and experimental values of dipole moments reported by South and Grant^47^ is remarkably high (see Figure 7). Notably, GruPol accurately reproduces the 10 au difference between dipole moments of the two myoglobin species. Moreover, our results show a similar trend to recent reports on electrostatic properties of SW myoglobin,^38^ where a maximum of dipole moment was also determined to be around pH 7.5. At the same time, those studies overestimate the prediction of the dipole moment at the isoelectric point by nearly two times which is not the case with our current approach, which uses precise values for core dipole moments. With regard to dipole moments that originates from charged functional groups on the protein’s backbone, GruPol utilizes a set of p*K*_a_ values (see the Supporting Information, Subsection S6.2) tailored for myoglobin molecules,^38^ which closely resemble the p*K*_a_ values of the individual amino acids. Interestingly, although fluctuations on p*K*_a_ values may affect severely the total dipole moments,^38^ in our study it appears to have minimal impact on the overall results after molecular dynamics simulation, likely due to the broad distribution of dipoles within multiple conformers. However, further investigations are necessary to fully understand the effects of varying the p*K*_a_ values on the database performance. Numerical quantities of dipole moments and polarizabilities for both myoglobin proteins can be found in the Supporting Information (Subsection S6.6).

### Role of H-Bond on Accurate Predictions

Being aware of the fact that the formation of intramolecular hydrogen bonds in macromolecules may significantly change electric response of the species,^48^ we also conducted investigation to discern the influence of diverse intramolecular interaction environment on dipole moment and ESP response. Given the substantial prevalence of peptide bonds (occurring 129 times) and terminal groups (occurring 28 times) among the peptides used to create GruPol’s entries, a comprehensive scan for hydrogen bonds was incorporated into the analysis. It is important to note that the scan covers not only intramolecular hydrogen bond formation but also those formed via solvation. These hydrogen bonds are significant for the recognition of specific building blocks, given their capacity of intrinsically changing the protein conformation, as discussed below.

To simplify our studies, peptide bonds involved in hydrogen bond formation were divided into two groups: −CO acceptors and −NH donors. Then clustering was performed based on the hydrogen bond D–H···A angle, whereby, due to the above-mentioned two subgroups, two cases are considered. The angle either involves the −CO group as an acceptor and N–H, O–H, or S–H groups as donors or −NH group as a donor and N, O, and S atoms as acceptors. No weaker hydrogen bonds involving C–H groups were considered. Because of the existing constraints regarding the number of building blocks, a distance threshold of 2.5 Å between the hydrogen and acceptor atoms was assumed for the peptide bond scan, thereby omitting the simultaneous differentiation of angle and distance. Angles seem to be more crucial when distinguishing, for example, between various protein secondary structures, and anyway the chosen distance threshold is wide enough to consider all hydrogen bonds that a peptide bond could form. Notably, the influence on those groups involved in multiple H-bonds, although less frequent than single occurrences, was accounted for by averaging the angles.

**Figure 8:**
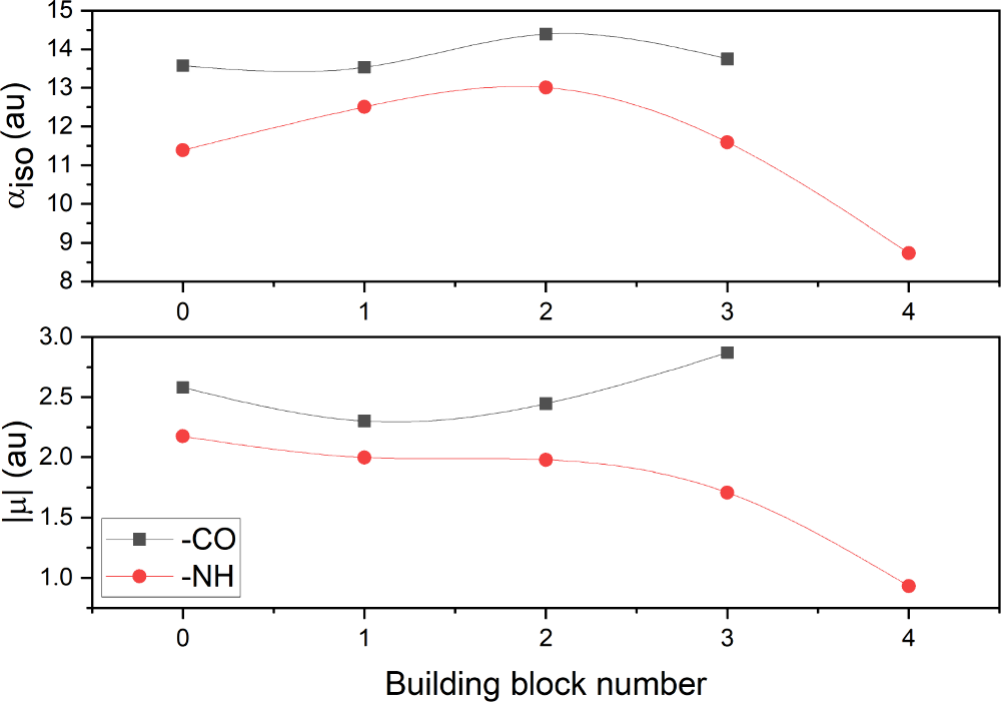
Variation of polarizability (top) and dipole moment (bottom) depending on HNC(O···H–D) or (A···H–N)CO angle when a peptide bond is involved in an intramolecular hydrogen bond. A, D = S, O, N. The building block number refers to the absence of H-bonds (−CO-0 and −NH-0) and angles within the range of 85°–112°, 112°–120°, 120°–135°, and 135°–180° (−NH-1, −NH-2, −NH-3, and −NH-4, respectively) and 70°–92°, 92°–130°, and 130°–180° (−CO-1, −CO-2, and −CO-3, respectively).

**Figure 9:**
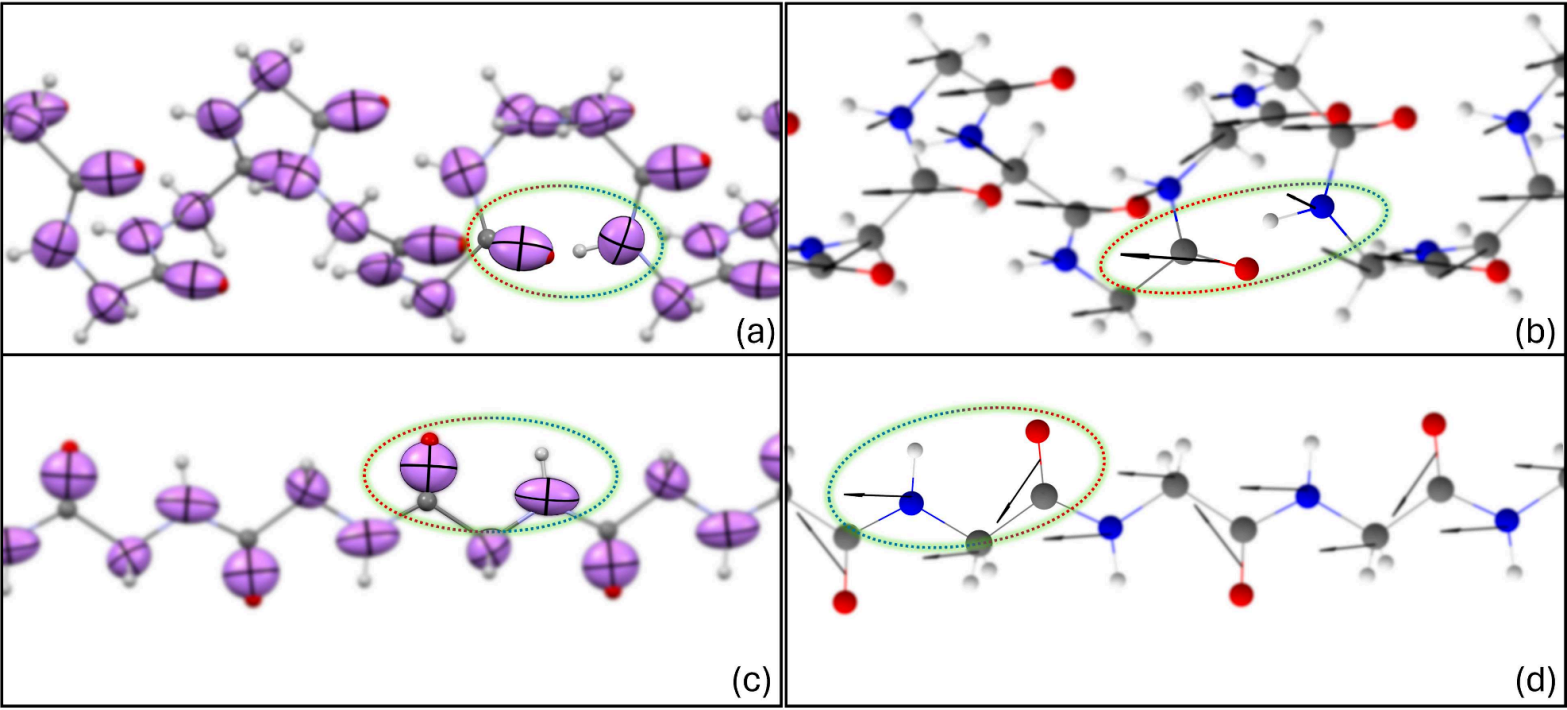
Differences in polarizabilities and dipole moments of peptide bonds when their constituent building blocks are involved in helix (a, b) and sheet arrangements (c, d), respectively.

In the context of peptide bonds, H-bond angles below 90° involving the CO group and below 120° for the NH group indicate the presence of more parallel arrangements between D–H and A–R groups, whereas angles higher than approximately 130° suggest the formation of more linear structures. Parallel linkages are associated with the formation of sheets, whereas linear hydrogen bonds are indicative of helix within the secondary structure.^49^ Since the database was primarily built employing peptides with 4–5 residues, the occurrence of parallel hydrogen bonds is more prominent due to peptide size limitation for helix formation. Furthermore, it is worth noting that angles around 120° typically involve the formation of two hydrogen bonds, although less frequent than the formation of a single H-bond. More details regarding the H-bond distribution and associated quantities can be found in the Supporting Information, Section S4. These findings underscore the significance of accounting for hydrogen bonds when precise determination of electric moments of peptide bonds is desired. Since these are strong intermolecular interactions, they can be responsible for an extensive polarization of the electron density of the involved building blocks.^48^ Moreover, it is evident that the NH group is highly responsive to the orientation of the N–H···O hydrogen bond, impacting both the polarizabilities and dipole moments. On the other hand, variations on those properties among CO groups, based on different H-bond arrangements, are less prominent but still discernible, as shown in Figure 8. Figure 9 depicts the differences in polarizabilities as well as dipole moments between helix and sheet arrangements, respectively, in a peptide fragment. The influence of H-bonds becomes evident as the orientation of ellipsoids and dipole vectors adjust, extending toward bonding formations. This highlights that although there might not be substantial discrepancies in the isotropic values, the shape of the tensor can experience significant alterations. Individual components of dipole moments and polarizability tensors are given in the Supporting Information, Section S4.

Given the limitations imposed by the number of conformers available during the studies and the complexity of clustering approaches, we decided to further constrain our analysis of terminal groups. Therefore, we created two groups for each −NH_3_^+^ and −COO^–^ considering only the presence or not of the H-bond, without any further geometrical considerations. Our assumption is that if hydrogen bond is formed on only one O atom of the COO^–^ group, it comprises the first subgroup, CTER_1_, whereas when the COO^–^ group forms two hydrogen bonds, it would result in a similar geometry as having no hydrogen bonds, thus belonging to the second subgroup, CTER_2_. For the −NH_3_^+^ group, the first subgroup represents the case where only one H atom forms a hydrogen bond (NTER_1_), while the second subgroup encompasses situations where more than one terminal H atom is involved in H-bond, or none (NTER_2_). It is important to note that for both −COO^–^ and −NH_3_^+^ functional groups, the presence of only one hydrogen bond predominates. When a building block participates in multiple H-bonds, it facilitates a more effective distribution of charges, thereby reducing the overall dipole moment. Additionally, polarizabilities are also affected by restricting the volume of the building blocks, potentially resulting in a decrease in this property as well. Table 1 summarizes polarizabilities and dipole moments calculated for the four above-mentioned building blocks.

**Table 1:** Differences in Polarizabilities and Dipole Moments for Different Terminal Groups Building Blocks

ine group	μ (au)	α_iso_ (au)
ine CTER_1_	2.9108	24.4074
CTER_2_	2.6014	24.1232
NTER_1_	1.4740	9.9702
NTER_2_	1.3104	10.2273
ine		

In the case of the −COO^–^ group, the polarizabilities and dipole moments exhibit an increase when a single hydrogen bond is present, reaching a value nearly 50% higher in the most extreme scenario. Conversely, the −NH_3_^+^ group experiences an increase in dipole moment but a decrease in polarizabilities when involved in a single hydrogen bond. This behavior mirrors the characteristics observed in parallel hydrogen bonds involving the peptide connection.

## Conclusion

In this paper we introduced GruPol software (available at https://www.polaber.eu/) designed for estimation of molecular and building block polarizabilities, dipole moments, and electrostatic potentials of biomolecules, including complex macromolecules. With a comprehensive collection of approximately 100 building blocks, encompassing 20 natural amino acid residues, GruPol demonstrates remarkable accuracy in predicting the above-mentioned properties, despite the size of the considered system. The achieved error rates are of less than 10%–15% for both polarizabilities and dipole moments. The high precision of predicted properties not only is evident for isotropic values but also extends to all components of the tensors that describe them. Well-described anisotropy is a key point to correctly characterize subtle variations in the studied system resulting from conformational changes, hydrogen bond assemblies, and polarization of electron density upon formation of other chemical bonding.

Since the introduction of the techniques used in GruPol,^9−12^ a number of significant improvements were developed to better describe the electric and electrostatic properties of macromolecules. In particular, a hydrogen bond scan is implemented, which significantly improves the prediction of the magnitude and orientation of dipole moments as well as the anisotropy of polarizability tensors. Moreover, the accuracy of the molecular electrostatic potential is significantly enhanced, allowing the splitting of functional group vectors and reassembling atomic contributions for peptide bonds and terminal building blocks. Additionally, the database is able to correctly grasp changes of the electric moments when the acidicity, i.e., pH of the aqueous environment of the macromolecule, changes. Our pH simulations yielded remarkable agreement with experimental results reported for myoglobin species. It is also important to point out that to the best of our knowledge, this study reports for the first time a full description of atomic, group, and molecular polarizabilities of the myoglobin proteins. Therefore, we emphasize here once again the uniqueness of the GruPol database in predicting polarizabilities of large systems and individual functional groups with a complete tensorial description (i.e., all diagonal and off-diagonal components are given), thus accounting for anisotropy, so often overlooked when estimating electric moments and electrostatic properties. Looking ahead, our future plans involve introducing more residues into GruPol, expanding the database beyond proteins and linear properties, to encompass general molecules and metal ions. We aim at the development of new entries for various prosthetic groups, with special attention to porphyrins rings and metal ions such as Fe^2+^/Fe^3+^, Zn^2+^, Cu^1+^/Cu^2+^, Mn^2+^, and Co^2+^. Some of our and others preliminary studies on organometallic complexes^50,51^ already showed promising results in the determination of atomic polarizabilities of metal centers, where not only the symmetry of the coordination polyhedron is well-reflected but also some optoelectronic properties (e.g., refractive index) are well-reproduced. This progressive expansion will enhance the versatility and applicability of GruPol, also expanding the range of properties to nonlinear ones, making it an invaluable resource for researchers across various domains of molecular science. We also believe that introducing anisotropy of polarizability to the system may be beneficial to the polarizable force field methods where the more precise introduction of the anisotropy of polarizability is expected to provide better insight into the structure–property correlation and virtual design of novel materials.

## Supporting Information Available

Additional information on data storage, using GruPol, visualizing results and software add-ons; building blocks definition and H-bond clustering; splitting methodology for ESP calculations; pH dependencies on dipole moments and polarizabilities; database validation and statistics (PDF)
