# Supplementary material for: Unveiling GruPol: Predicting Electric and Electrostatic Properties of Macromolecules via the Building Block Approach
Source: J Phys Chem B. 2024 Jul 8;128(33):7954–65. doi: 10.1021/acs.jpcb.4c03062 (PMC11345817; doi:10.1021/acs.jpcb.4c03062)
Supplement: Supplementary file 1 — jp4c03062_si_001.pdf [file jp4c03062_si_001.pdf]

Supporting Information for:

Unveiling GruPol: Predicting Electric and Electrostatic Properties of Macromolecules via Building Block Approach

Raphael F. Ligorio,[a] Paul Grosskopf,[a] Leonardo H. R. Dos Santos,[b] Anna Krawczuk*[a]

[a] Institute of Inorganic Chemistry, University of Goettingen, Tammannstrasse 4, D-37077 Göttingen, Germany; E-mail: *anna.krawczuk@uni-goettingen.de

[b] Departamento de Química, Universidade Federal de Minas Gerais, Av. Pres. Antônio Carlos 6627, 31270-901 Belo Horizonte MG, Brazil

Contents

S1 Data storage information **3**

S2 Using GruPol, visualizing results and solid state properties **4**

S3 Building blocks **5**

S4 H-bond clustering **11**

S5 Electrostatic Potential **13**

S6 pH Dependencies **14**

S6.1 Water box cutoffs 14

S6.2 Charged residues pKa 15

S6.3 Dipole moments corrections 16

S6.4 Polarizabilities corrections 16
S6.5 Dipole moments predictions 18
S6.6 Myoglobin and Alanine oligomers in water 18

S7 Database validation **21**

S8 Building blocks entries and statistics **23**

S1 Data storage information

Working version of GruPol database together with molecular geometries used in this paper and PolaBer output testing files are stored as a separate dataset in GöttingenResearchOnlineData platform. See 10.25625/0LSWU4 which contains the folders below:

TEST FOLDER 1: Contains a PDB file for running GruPol (atom labels must follow CHARMM force field).

TEST FOLDER 2: Contains a PolaBer output file for running CrysPol. The given CIF file provides the necessary information concerning crystal symmetry for testing.

PROTEINS VALIDATION COORDINATES: Contains the coordinates of the molecules used to benchmark GruPol.

DYNAMICS COORDINATES: Contais the coordinates for the HH and SW myoglobins, as well as the Alanine oligomers obtained via molecular dynamics.

TEST CHARGE COORDINATES: Contains the coordinates of the three peptides used for testing the charged model in the three different protonation states.

S2 Using GruPol, visualizing results and solid state properties

To enhance usability and visualize the properties, GruPol offers a user-friendly graphical interface. Through this interface, users can easily generate an input file for GruPol by simply placing a .pdb file in the working folder. This interface allows users to select various parameters, including the run mode, solvent, pH, radial cutoff for the dipole interaction model, convergence criteria, number of cycles, resolution for the electrostatic potential, and the option to create new entries in developer mode.

In essence, the ”run mode” keyword enables users to initiate the code from specific checkpoints in case of an error detection. Currently, only run mode zero is available. This mode encompasses all the modules, such as extracting building blocks from a .pdb file, aligning and fitting these building blocks into a specified protein geometry, generating electrostatic potential maps, and, if desired, estimating solvent effects.

The supplementary keywords in the interface serve distinct purposes. ”Solvent” enables users to specify the presence of a solvent, currently restricted to water. The ”pH” parameter simulates charges on the protein's backbone by setting the solution's pH. ”Radial cutoff” facilitates the creation of an ellipsoid around the protein, emulating an isotropic environment based on the protein's dimensions. ”Convergence criteria” defines the acceptable variation threshold between consecutive ADIM iterative cycles, signifying the point of convergence. ”Number of cycles” sets the maximum iteration count. The ”Electrostatic potential resolution” determines the spatial grid's point separation, exclusively available for .pdb files lacking solvent molecules. Furthermore, ”developer mode” allows for new entry generation, necessitating a modified .pdb file with atomic dipole moments and polarizabilities of a designated protein, from which building blocks are extracted to generate these new entries.

The current database exclusively accommodates neutral residues, implying that the amino acid residues should not carry a charge (nevertheless, charges can be simulated changing the pH value). An exception is made for the terminal groups, necessitating the protein to be in its neutral zwitterion state. Any additional hydrogen atoms in basic residues, resulting in charges on lysine and arginine residues, for example, are disregarded during the identification of building blocks. If hydrogen atoms are absent in aspartic and glutamic acid residues, the program automatically appends a hydrogen atom to create the inertia tensor, thereby ensuring the correct rotation of the building

block. Furthermore, GruPol interface offers a convenient link to access PolaBer directly. Additionally, it provides a package named as CrysPol, designed for simulating solid-state properties, including refractive indices, electric susceptibility, and dielectric constants. CrysPol utilizes the output file of PolaBer, which contains atomic dipole moments and polarizabilities, to rotate a specified molecule or asymmetric unit. This rotation reproduces a crystal supercell, and the environment is taken into account through dipole interactions. This transition from the gaseous to the solid phase is thus achieved through the implementation of ADIM.

Lastly, we provide the option to visualize atomic and functional group dipole moments and polarizabilities, generated through GruPol and CrysPol. This is possible with the VisPol package, which allows users to visualize visualize those properties. It is important to note that for larger molecules, the picture generation process in VisPol can be time-consuming. Nevertheless, it serves as a valuable tool for results assessment when dedicated visualization software is lacking. Figure S1 offers a brief glimpse of the GruPol interface.

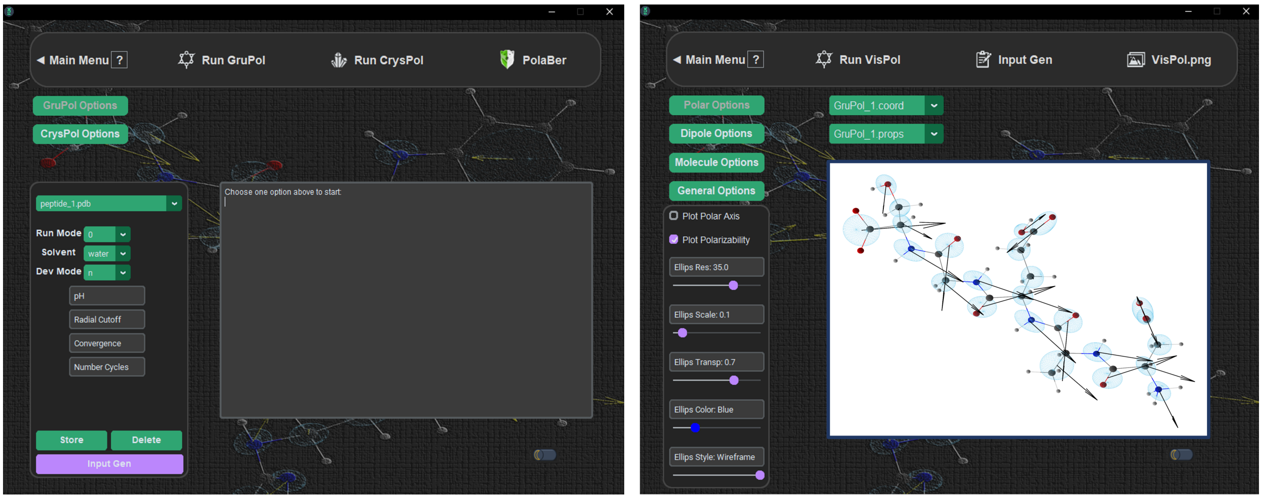

Figure S1: Introducing GruPol interface. On the left, options to control the properties btained using the database, showing a direct link to PolaBer and CrysPol. On the right it s shown functional groups dipole moments and polarizabilties obtained via GruPol/VisPol

S3 Building blocks

Table S1: Atom labels of each amino acid building block present within GruPol. The numbers in parentheses denote the use of higher atomic number (charges) for constructing the charge tensor. These elevated charges serve exclusively for establishing the orientation of the local coordinate system. The positions of individual atoms within the amino acid residues are depicted in Figure S2. To align each building block, we employ CHK atoms, which must reside in the positive octant post-rotation. Dummies are utilized solely for the purpose of constructing the inertia tensor and do not impact the properties of the building blocks. For the NTER group, a dummy atom is positioned where a hydrogen atom involved in an H-bond exists (designated as HTn, with 'n' being either 1, 2, or 3). In cases where no H-bond is present or multiple H-atoms are involved in an H-bond, the dummy atom is placed at the position of HT1. Regarding the CTER group, if no H-bonds involve the oxygen atoms within this group or if both oxygen atoms participate in H-bonds, no dummy atoms are introduced. However, if only one oxygen atom is engaged in H-bonds, a dummy with a charge of 32 a.u. is added at the position of this atom (designated as OTm). Yet, a dummy atom (OTn) with a charge of 16 a.u. is introduced at the position of the O-atom not involved in H-bond.

Building-Blocks	CHK	ATOM	Dummies
NTER	N	HT1 HT2 HT3	HTn(5) CA(12)
CTER	C	OT1 OT2	OTn(16) OTm(32)
ALA_ZWI	CA	HA, CB, HB1, HB2, HB3	N(14), C
GLY_ZWI	CA	HA1, HA2	N, C
SER_ZWI_1st	CA	HA	N(14), C, CB(12)
OH_ZWI_SER	HG1	OG	CB
SER_ZWI_2nd	CB	HB1, HB2	CA, OG
HSD_ZWI_1st	CA	HA	N(14), C, CB(12)
HSD_ZWI_2nd	CB	HB1, HB2	CA(12), CG
IMI_ZWI	CG	CD2, CE1, ND1, NE1, HD1, HD2, HE1	CB
TYR_ZWI_1st	CA	HA	N(14), C, CB(12)
TYR_ZWI_2nd	CB	HB1, HB2	CA(12), CG
PHENOLIC_ZWI	CZ	CG, CD1, CE1, CD2, CE2, HD1, HE1, HD2, HE2	OH, HH
OH_ZWI_PHE	HH	OH	CZ
GLU_ZWI_1st	CA	HA	N(14), C, CB(12)

Table S1: Coninuation.

Building-Blocks	CHK	ATOM	Dummies
GLU_ZWI_2nd	CB	HB1, HB2	CG, CA(12)
GLU_ZWI_3rd	CG	HG1, HG2	CD, CB(12)
CO_ACID_ZWI	CD	OE1	OE2(16)
OH_ACID_ZWI	HE2	OE2	CD
PROT_ASPARTIC_ACID_GROUP_1st	CA	HA	N(14), C, CB(12)
PROT_ASPARTIC_ACID_GROUP_2nd	CB	HB1, HB2	CG, CA(12)
CO_ACID_ZWI	CG	OD1	OD2(16)
OH_ACID_ZWI	HD2	OD2	CG
ASN_ZWI_1st	CA	HA	N(14), C, CB(12)
ASN_ZWI_2nd	CB	HB1, HB2	CG, CA(12)
ASN_ZWI_3rd	CG	OD1	ND2(14),CB(12)
ASN_ZWI_4th	ND2	HD21, HD22	CG, OD1
THR_ZWI_1st	CA	HA	N(14), C, CB(6)
THR_ZWI_2nd	CB	HB	CA, OG1
THR_ZWI_3rd	CG2	HG21 HG22 HG23	CA CB HB(8)
OH_ZWI_THR	HG1	OG1	CB
PRO_ZWI_1st	N	O, C	CA(12)
PRO_ZWI_2nd	HA	CA, CB, CG, CD HB1, HB2, HG1, HG2, HD1, HD2	–
CYS_ZWI_1st	CA	HA	N(14), C, CB(12)
CYS_ZWI_2nd	CB	HB1, HB2	SG, CA
CYS_ZWI_3rd	S	HG1	CB
MET_ZWI_1st	CA	HA	CB(12), C, N(14)
MET_ZWI_2nd	CB	HB1, HB2	CA(12), CG

Table S1: Coninuation.

Building-Blocks	CHK	ATOM	Dummies
MET_ZWI_3rd	CG	HG1, HG2	SD, CB
MET_ZWI_4th	SD	–	CG, CE(12)
MET_ZWI_5th	CE	HE1, HE2, HE3	SD, CG
ILE_ZWI_1st	CA	HA	N(14), C, CB(12)
ILE_ZWI_2nd	CB	HB	CA
ILE_ZWI_3rd	CG2	HG21, HG22, HG23	CA, CB, HB(2)
ILE_ZWI_4th	CG1	HG11, HG12	CD, CB(12)
ILE_ZWI_5th	CD	HD1, HD2, HD3	CB, CG1, HG11(2)
VAL_ZWI_1st	CA	HA	N(14), C, CB(12)
VAL_ZWI_2nd	CB	HB	CA, CG1(12), CG2(16)
VAL_ZWI_3rd	CG1	HG11, HG12, HG13	HB(2), CA, CB
VAL_ZWI_4th	CG2	HG21, HG22, HG23	HB(2), CA, CB
GLN_ZWI_1st	CA	HA	N(14), C, CB(12)
GLN_ZWI_2nd	CB	HB1, HB2	CG, CA(12)
GLN_ZWI_3rd	CG	HG1, HG2	CD, CB(12)
GLN_ZWI_4th	CD	OE1	NE2(14), CG(12)
GLN_ZWI_5th	NE2	HE21, HE22	CD, OE1
LEU_ZWI_1st	CA	HA	N(14), C, CB(12)
LEU_ZWI_2nd	CB	HB1, HB2	CA(12), CG
LEU_ZWI_3rd	CG	HG1	CB
LEU_ZWI_4th	CD1	HD11, HD12, HD13	CB, CG, HG(2)
LEU_ZWI_5th	CD2	HD21, HD22, HD23	CB, CG, HG(2)
ARG_ZWI_1st	CA	HA	N(14), C, CB(12)

Table S1: Coninuation.

Building-Blocks	CHK	ATOM	Dummies
ARG_ZWI_2nd	CB	HB1, HB2	CG, CA(12)
ARG_ZWI_3rd	CG	HG1, HG2	CB, CD(12)
ARG_ZWI_4th	CD	HD1, HD2	NE
ARG_ZWI_5th	NE	HE	CD(12), CZ
ARG_ZWI_6th	NH1	HH11	CZ
ARG_ZWI_7th	NH2	NH21, NH22, CZ	NE, NH1(14)
LYS_ZWI_1st	CA	HA	N(14), C, CB(12)
LYS_ZWI_2nd	CB	HB1, HB2	CA(12), CG
LYS_ZWI_3rd	CG	HG1, HG2	CD, CB(12)
LYS_ZWI_4th	CD	HD1, HD2	CG(12), CE
LYS_ZWI_5th	CE	HE1, HE2	NZ
LYS_ZWI_6th	NZ	HZ1, HZ2	CE
TRP_ZWI_1st	CA	HA	N(14), C, CB(12)
TRP_ZWI_2nd	CB	HB1, HB2	CA(12), CG
TRP_ZWI_3rd	CG	CD1, HD1	CB, CD2(12)
TRP_ZWI_4th	NE1	HE1	CD1, CE2(12)
TRP_ZWI_5th	CE2	CD2, CE3, CZ3 CZ2, CH2, HE3, HZ3, HH2, HZ2	NE1
PHE_ZWI_1st	CA	HA	N(14), C, CB(12)
PHE_ZWI_2nd	CB	HB1, HB2	CG, CA(12)
PHE_ZWI_3rd	CG	CD1, CD2, CE1, CE2, CZ, HD1, HD2, HE1, HE2, HZ	CB, CA(1)

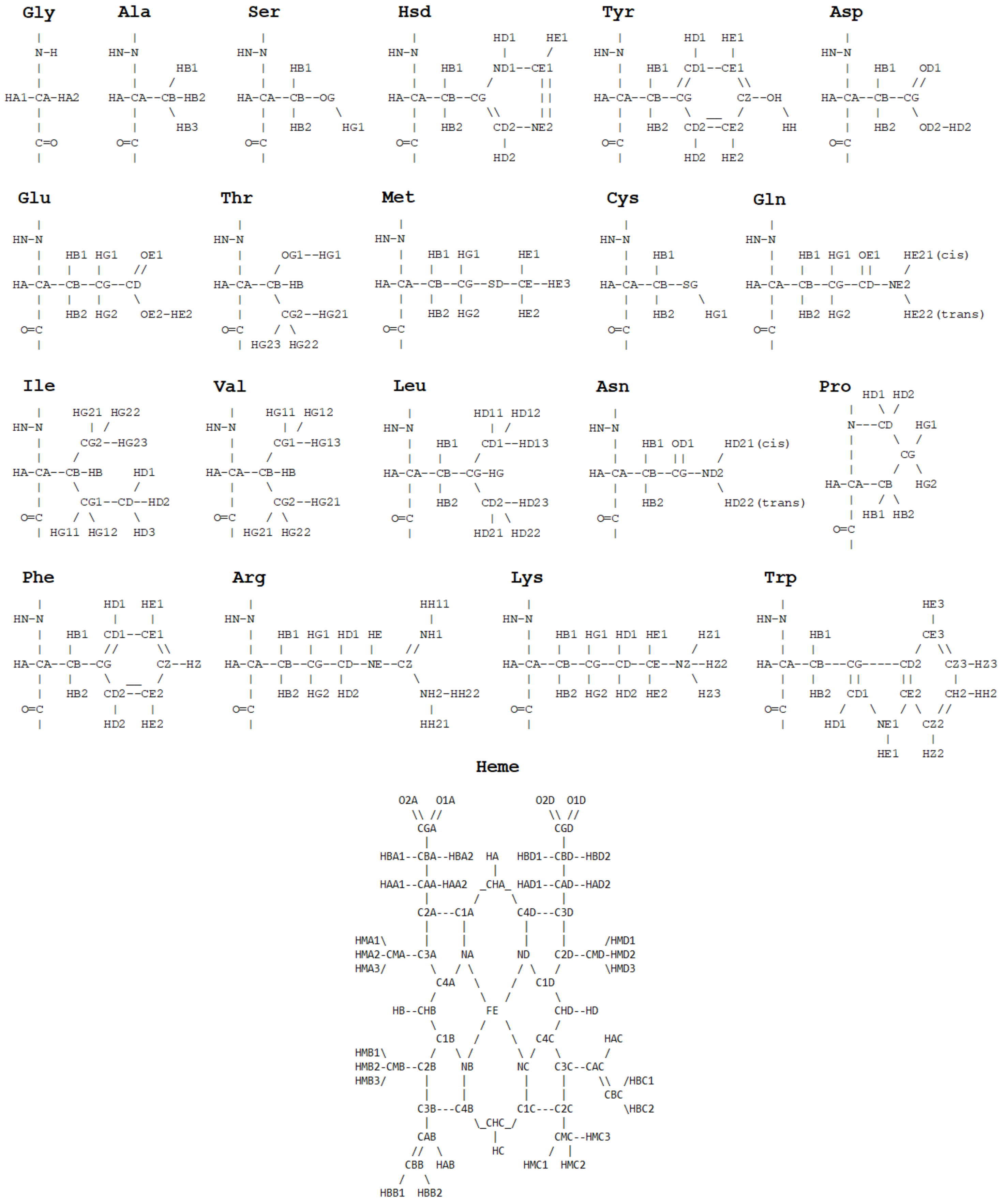

Figure S2: Amino acid residues and the heme group with their respective atom labels, in agreement with the CHARMM force field.

S4 H-bond clustering

Table S2: Polarizabilities and dipole moments for different peptide bonds building blocks according to the angle HNC(O...H-D) and (A...H-N)CO (A, D=S, O, N). Dipole moments and polarizabilities are given in atomic units.

	Angle (^{o})	μ_x	μ_y	μ_z	α_{xx}	α_{yy}	α_{zz}	α_{xy}	α_{xz}	α_{yz}
NH_0	-	-1.1550	1.8409	0.0420	13.9729	14.3338	6.1670	-6.0405	-0.2124	-0.1975
NH_1	85-112	-1.0254	1.7142	0.0346	14.3919	17.3008	5.8652	-7.7067	0.6252	0.0401
NH_2	112-120	-1.2680	1.5194	0.0110	15.3680	17.9589	5.8272	-8.0883	1.0207	0.4096
NH_3	120-135	-0.6469	1.5796	0.0599	13.7613	15.4323	5.6941	-3.5787	0.3747	0.8650
NH_4	135-180	0.2946	0.8787	0.0855	14.1801	12.0072	5.5846	-1.1718	0.4228	0.7696
CO_0	-	-1.1247	2.3206	0.0660	15.5968	18.5683	7.0521	-1.1232	0.0267	0.4592
CO_1	70-92	-0.7982	2.1577	0.0484	15.7231	17.6890	6.5562	1.5937	0.6914	0.0336
CO_2	92-130	-1.2795	2.0826	0.0226	15.5527	19.7011	7.1852	0.1073	0.1613	-0.3367
CO_3	130-180	-2.0562	1.9970	0.1601	18.6463	16.4267	7.9190	-5.7336	0.5256	0.1830

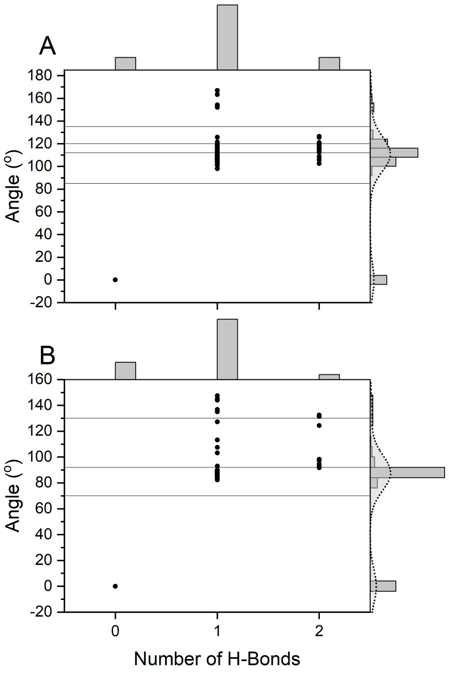

Figure S3: Distribution of peptide bonds with regard to the presence of hydrogen bonds and the angle (A...H-N)CO, top and HNC(O...H-D), bottom. The 'Number of H-Bonds' corresponds to the number of H-bonds detected for each building block. In instances where two hydrogen bonds were identified, the average angle value is considered. The main text provides information on the threshold used to detect the presence of hydrogen bonds, along with additional details.

S5 Electrostatic Potential

The vector corresponding to the COO^{-} group was divided under the assumption that the C-atom's dipole moment aligns with the group's dipole moment, with a scaling factor of 0.3 applied. Regarding the O-atoms' vectors, we initially computed the unitary vector parallel to the C-O bond, as illustrated by the orange arrows in Figure S4. The group's dipole moment approximately splits the O-C-O angle in half, leading us to assume that both oxygen atoms possess identical dipole moment magnitudes. If these vectors are of equal magnitude, their combination forms a diamond shape, with a diagonal length constituting 70% of the group's dipole moment, given that we have already scaled the C-atom dipole moment. In an ideal scenario, the summation of the split vectors should align precisely with the group's dipole moment. However, our simplification assumes an exact bisecting of the O-C-O angle. Consequently, the summation of the three vectors does not precisely yield the group's dipole moment. Nevertheless, the errors can be neglected, and this approach has consistently proven to significantly enhance GruPol's predictions. Noteworthy, the scale factors used were based on fitting the split dipole moment in respect to atomic dipole moments of the Gly-Gly dimer.

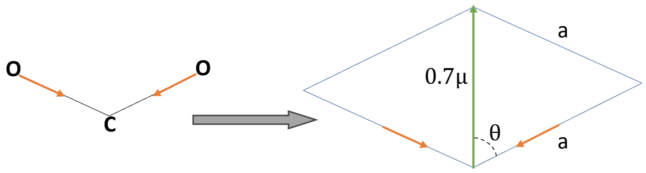

Figure S4: The dipole moment splitting method for the COO^{-} group, using the cosine law, where 'a' (blue lines) represents the magnitude determined based on the scaled module of the group's dipole moment (0.7μ) and half of the O-C-O angle (θ). The unitary vector connecting O-C was resized to match the magnitude of 'a'

In the context of the NH(CO) group (found in peptide bonds), our assumption was that the O-atom dipole vector aligns itself toward the C-atom. Remarkably, we observed that, for the Gly-Gly dipeptide, the C-atom dipole points towards the center of mass of the HNCO group. Therefore, knowing the directions of both C and O-atom dipole moments, we scale these vectors, thus obtaining a parallelogram in which the diagonal represents the group's dipole moment. This calculation ensures that the summation of the two split vectors yields precisely the group's dipole moment.

S6 pH Dependencies

S6.1 Water box cutoffs

In order to estimate GruPol corrections, an ellipsoid was obtained from the water box fitting the edges of the proteins, plus a radial criteria of 6 a.u.. A scheme of how the ellipsoid is obtained is given in the Figure S5.

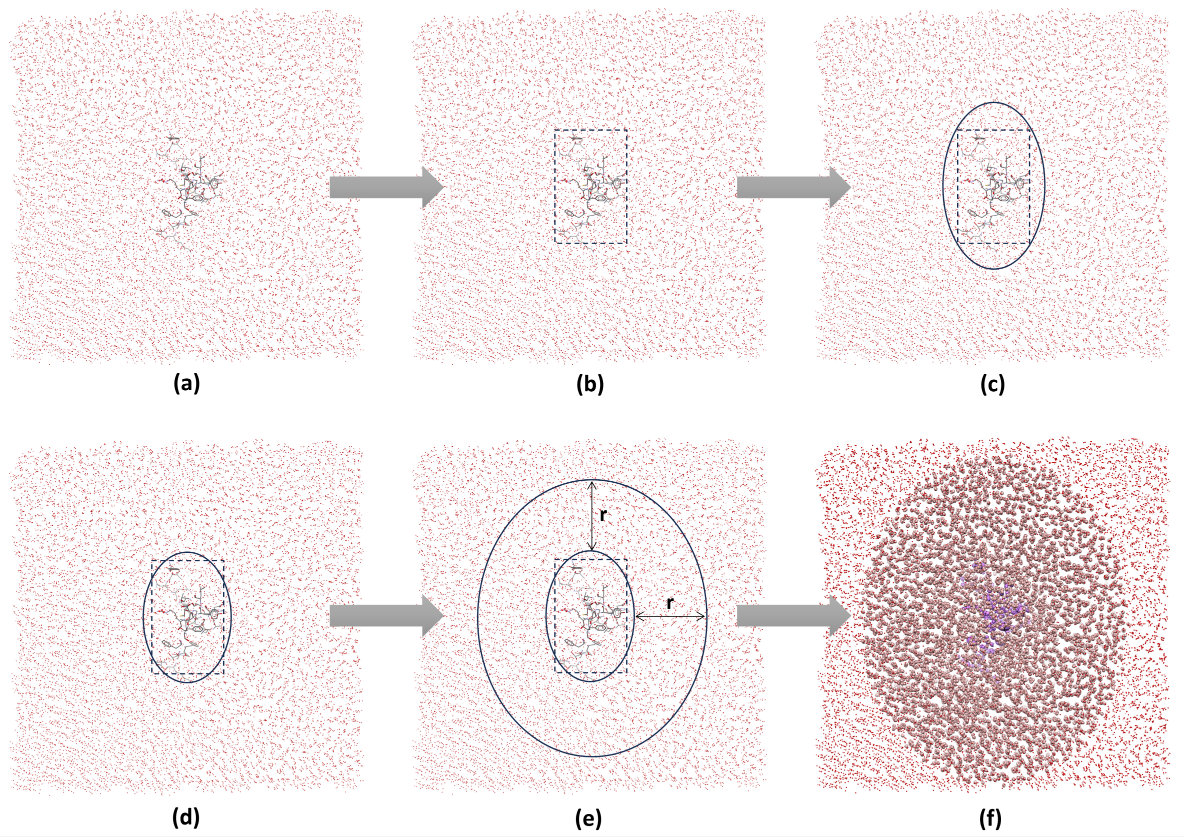

Figure S5: A random protein in a water box (a). A box with origin in the center of the protein is obtained (b) followed by circumscribing it with an ellipsoid (c). The ellipsoid is then shrinked keeping the same ratio of the main axis until its minimum size that contains all the protein (d). A radial criteria, r, is applied according to the user demand (e). An example is given in (f)

S6.2 Charged residues pKa

The charge of a given titratable residue is given by the Handerson-Haselbach model, expressed by the Equation S1, for acid groups and Equation S2 for basic residues. Charges are added in the center of "charges" (atomic numbers) of a given titratable building block, and not at the centre of the entire residue.

$$-q = \frac{10^{pH-pKa}}{1-10^{pH-pKa}} \tag{S1}$$

$$+q = \frac{1}{1-10^{pH-pKa}} \tag{S2}$$

The values of pKa used are given in the Table S3

Table S3: pKa values used to calculate charges of titratable residues.

Group	Charge	pKa
ACID	-	4.4
CYS	-	8.5
TYR	-	10.1
ARG	+	12.5
LYS	+	10.6
HIS	+	6.6
CTER	+	4.0
NTER	-	8.0

It is important to note that the terminal groups exhibit a charge with a counterintuitive sign due to their previous classification as charged groups during the creation of the database entries. This approach is designed to achieve a balanced charge state for the zwitterionic molecule. For these groups, we calculate the expected charge at a given pH and adjust it, assuming an initial zwitterion molecule with a charge of +1 for the NTER group and -1 for the CTER group. Therefore, if at a given pKa, the charge of the NTER group is +0.8 instead of the initial value of +1, we apply a compensating charge of -0.2 to reach the desired value.

S6.3 Dipole moments corrections

The distribution of charges on the protein's backbone of generates a dipole moment, known as the charge dipole moment. Additionally, the dipole moment associated with the electron density polarization due to chemical bonding is referred to as core dipole moment. The total dipole is the sum of the charge and core contributions.

$$\vec{\mu}_{total} = \vec{\mu}_{core} + \vec{\mu}_{charges} \tag{S3}$$

where $\vec{\mu}_{charges}$ is given by:

$$\vec{\mu}_{charges} = \frac{1}{2}(|q_{pos}| + |q_{neg}|)\vec{R} \tag{S4}$$

where $\vec{R}$ is the vector with origin at the center of the negative charges pointing to the center of positive charges, given by:

$$\vec{R} = \frac{1}{q_{pos,total}} \sum |q_{pos,i}|\vec{r_i} - \frac{1}{q_{neg,total}} \sum |q_{neg,j}|\vec{r_j} \tag{S5}$$

where $\vec{r}$ is the position of each building block within each i or j titratable residue, with positive or negative charges respectively. Yet, the core dipole moment is also corrected using the electric field $\vec{F}$ generated by the presence of the charges.

$$\vec{F_k} = \frac{q}{r^2}\hat{r} \tag{S6}$$

where $|r|$ is the module of the vector connecting a given charge to a given building block. $\hat{r}$ is an unitary vector with origin at the charge, pointed to a given building block, p. The total electric field experienced by a building block is the summation of each the individual electric field $\vec{F_k}$.

$$\vec{\mu}_{\text{core},p} = \vec{\mu}_p^0 + \alpha_p \sum \vec{F_k} \tag{S7}$$

where α_p is the polarizbility of a given building block p.

S6.4 Polarizabilities corrections

As reported in previous studies[1], the presence of charges on the protein's backbone does not significantly impact either the molecular polarizability or the values of its constituent building blocks,

[1] A. Jabluszewska, A. Krawczuk, L. H. R. Dos Santos, P. Macchi, ChemPhysChem 2020, 21, 2155.

Table S4: Polarizabilities obtained for three protonation states for each molecule at the M06-HF/cc-pVDZ level of theory.

1. Ala-Asp-Lys-Ala						
	α_{xx}	α_{yy}	α_{zz}	α_{xy}	α_{xz}	α_{yz}
All neutral	219.3	219.5	221.2	1.9	-4.9	6.2
Terminal zwitt.	222.6	223.4	226.7	-0.4	-7.2	4.0
All charged	225.0	226.0	227.1	0.5	-7.0	3.9

2. Arg-Glu-Ser-Gly						
	α_{xx}	α_{yy}	α_{zz}	α_{xy}	α_{xz}	α_{yz}
All neutral	243.9	222.5	238.4	-4.4	-20.3	5.4
Terminal zwitt.	246.7	224.7	243.4	-6.4	-20.5	5.0
All charged	241.3	223.9	251.5	-4.8	-22.5	5.1

3. Arg-Glu-Lys-Ser-Gly-Tyr-Ala						
	α_{xx}	α_{yy}	α_{zz}	α_{xy}	α_{xz}	α_{yz}
All neutral	532.6	399.3	456.6	14.5	4.5	-20.6
Terminal zwitt.	537.1	400.7	456.8	13.5	5.4	-23.3
All charged	549.8	405.9	471.7	16.2	4.7	-24.3

at least for small peptides. In our research, we have found that this observation holds true for larger peptides as well, which contain up to seven amino acid residues and six titratable groups. To further investigate, we constructed three peptides: 1. Ala-Asp-Lys-Ala, 2. Arg-Glu-Ser-Gly, and 3. Arg-Glu-Lys-Ser-Gly-Tyr-Ala. Afterwards, we calculated the polarizabilities for these peptides under three protonation states: all neutral residues, all charged residues, and the terminal zwitterion. Table S4 presents the polarizabilities calculated using Gaussian 16 software at the M06-HF/cc-pVDZ level of theory. The geometry of each molecule was optimized before quantum-mechanical calculations using the CHARMM force field.

Deviations in the main diagonal component were found to be as low as 3%. Furthermore, off-diagonal quantities remained consistent across different protonation states. Therefore, based on these observations, no adjustments or corrections were deemed necessary for polarizabilities due to

the presence of charges on the protein's backbone. However, more studies are demanded.

S6.5 Dipole moments predictions

The application of the proposed model to correct the dipole moment in various protonation states showed to be precise for all three tested peptides, as illustrated in Figure S6.

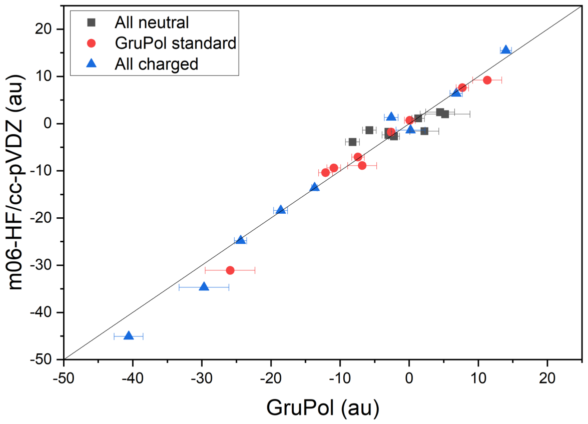

Figure S6: Cartesian components of dipole moments obtained using GruPol and at the M06-HF/cc-pVDZ level of theory. GruPol standard is the terminal zwitterion protonation state.

S6.6 Myoglobin and Alanine oligomers in water

The proposed approach has demonstrated its consistency when applied to three oligomers of alanine peptides and two myoglobin species: horse heart (HH) and sperm whale (SW), across various pH values. Regarding the alanine oligomers, the experimental data[2] lacks information about the pH at which the dipole moments were obtained. However, since these molecules only possess terminal charged groups, we anticipate that the measured pH is likely close to six, which corresponds to the isoelectric point based on the pKa values provided in Table S3. At this pH value, the dipole moments exhibited their highest values and a certain level of stability. This suggests that within the pH range of 5 to 7, variations in the dipole moment for all three molecules were not significant.

[2]P. J. Flory, P. R. Schimmel, J. Am. Chem. Soc. 1967, 89, 6807-6813.

Figure S7 and Table S5 present a comparison between the values obtained through GruPol and the experimental data.

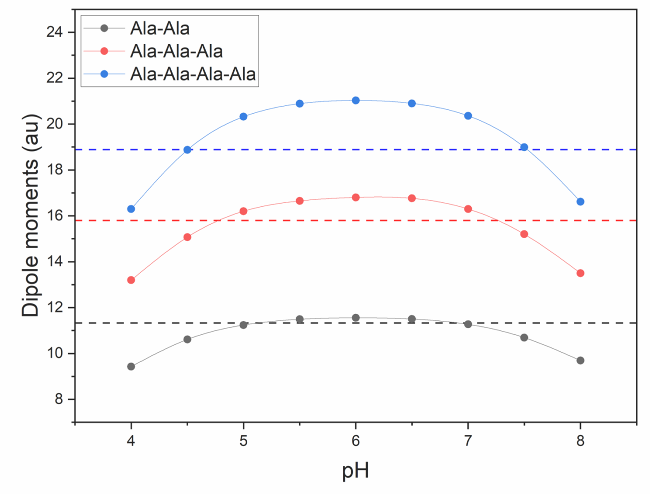

Figure S7: Comparison between GruPol results (at different pH) and experimental quantities (obtained from P. J. Flory, P. R. Schimmel, J. Am. Chem. Soc. 1967, 89, 6807-6813) for the three Alanine oligomers. Dashed lines represent experiment, whereas dots are related to GruPol results.

Table S5: Dipole moment and polarizabilities of two species of myoglobin estimated using GruPol and dipole moments obtained experimentally at different pH values. Error associated to GruPol refers to the standard deviation considering the values obtained for different conformers. Noteworthy, we do not present the polarizability in respect to pH since the database consider the same value for this quantity regardless the protonation state of a given residue.

Horse Heart		
pH	$\|\mu\|$ GruPol (au)	$\|\mu\|$ Exp (au)
5.5	63.5 ±11.0	57.4 ±5.7
6.0	66.7 ±11.0	65.4 ±6.5
6.5	71.9 ±11.0	70.4 ±7.0
7.0	78.1 ±12.0	74.0 ±7.4
7.5	81.4 ±12.0	76.7 ±7.7
α_{11} (au)	α_{22} (au)	α_{33} (au)
12254.4 ±83.5	12523.8 ±54.4	13038.5 ±83.1

Sperm whale		
pH	$\|\mu\|$ GruPol (au)	$\|\mu\|$ Exp (au)
6.5	54.2 ±15.0	61.4 ±6.1
7.3	62.7 ±16.0	63.3 ±6.3
8.0	64.7 ±16.0	64.5 ±6.4
α_{11} (au)	α_{22} (au)	α_{33} (au)
12377.5 ±112.5	12802.1 ±87.2	13218.5 ±70.6

S7 Database validation

Table S6: Dipole moments and polarizabilities of proteins calculated at the MO6HF/aug-cc-pVDZ and MO6HF/aug-cc-pVDZ level of theory and using GruPol. Values in atomic units. Atomic coordinates can be found in the folder "proteins_validation_coordinates", a subfolder inside GruPol's distribution, see https://doi.org/10.25625/0LSWU4.

MO6HF/aug-cc-pVDZ	μ_x	μ_y	μ_z	α_{xx}	α_{yy}	α_{zz}	α_{xy}	α_{xz}	α_{yz}
1a	-24.4	10.8	-19.3	866.1	858.9	844.9	-51.7	71.7	11.9
2a	-33.7	19.0	-27.5	969.3	887.7	950.6	-71.4	87.2	-13.6
3a	-15.1	5.4	-12.9	661.2	776.0	708.7	-43.2	66.2	-48.7
4a	-31.8	17.8	-27.0	901.0	837.6	964.8	-8.1	109.0	-63.5
5a	-37.0	18.6	-25.0	1016.4	879.2	959.7	-68.8	74.0	-71.6
6a	-23.8	7.7	-22.7	794.1	723.3	761.0	-19.7	75.3	-3.2
7a	-26.7	13.7	-15.1	958.8	827.9	762.7	-62.3	91.7	5.5
8a	-24.5	12.9	-19.0	734.4	640.7	685.1	-42.0	64.3	-9.4
9a	-23.6	5.2	-18.6	856.2	750.7	811.8	-9.4	78.2	-11.4
10a	-33.5	15.5	-24.6	1051.0	887.9	955.0	-80.9	92.3	-60.2
11a	-31.1	13.4	-26.2	816.5	666.2	858.6	-73.4	46.7	2.4
MO6HF/cc-pVDZ									
1a	-24.7	10.8	-19.5	751.3	762.6	740.1	-53.8	67.4	16.1
2a	-33.7	19.2	-27.7	852.3	778.0	839.6	-58.7	84.1	-6.3
3a	-15.3	5.5	-13.4	569.1	674.2	614.5	-43.8	62.1	-48.4
4a	-32.3	17.6	-27.2	783.4	736.5	848.0	-0.1	102.2	-57.9
5a	-37.3	18.8	-25.1	892.1	765.4	844.0	-61.4	63.9	-71.2
6a	-24.1	7.6	-22.9	701.2	624.8	668.5	-19.8	65.1	3.0
7a	-27.0	13.9	-15.1	855.1	728.2	650.3	-57.0	89.3	15.8
8a	-24.5	13.2	-19.1	648.4	563.0	598.4	-38.3	59.7	-6.2
9a	-23.8	5.1	-18.7	753.1	648.0	711.3	-5.2	70.7	-7.4
10a	-33.8	15.7	-24.4	923.0	769.1	840.2	-77.4	85.4	-57.6
11a	-31.3	13.3	-26.1	719.2	571.6	753.4	-76.3	42.6	8.8
GruPol									
1a	-19.6	13.8	-21.5	825.0	908.5	918.7	-31.3	14.4	6.5
2a	-32.1	18.4	-24.3	989.1	938.0	978.9	1.2	37.0	24.2
3a	-14.4	3.3	-13.7	702.7	774.0	734.8	-18.5	10.3	-20.6
4a	-28.6	18.1	-19.2	907.6	906.1	946.8	21.9	25.1	-12.4
5a	-37.9	17.8	-23.8	1019.6	898.9	971.5	-30.4	29.1	-42.9
6a	-19.2	8.3	-23.0	789.7	770.8	768.4	-3.4	2.0	-3.3
7a	-21.0	12.7	-16.0	958.4	846.5	790.2	-18.6	41.6	40.7
8a	-22.5	11.6	-19.1	770.2	660.0	688.2	-2.4	28.2	6.6
9a	-21.2	7.5	-19.2	858.0	779.4	807.8	18.6	49.3	11.7
10a	-30.4	11.8	-22.6	1021.0	957.2	980.0	-18.2	40.6	-34.3
11a	-31.9	15.3	-22.5	802.3	770.8	821.1	-62.0	7.5	25.6

Table S7: Dipole moments and polarizabilities of proteins calculated at the MO6HF/cc-pVDZ level of theory and using GruPol. Values in atomic units. Atomic coordinates can be found in the folder "proteins_validation_coordinates", a subfolder inside GruPol's distribution, see https://doi.org/10.25625/0LSWU4.

MO6HF/cc-pVDZ	μ_x	μ_y	μ_z	α_{xx}	α_{yy}	α_{zz}	α_{xy}	α_{xz}	α_{yz}
1b	-89.8	3.1	-58.2	1599.6	1128.3	1362.9	-48.1	252.1	-13.9
2b	15.7	42.8	-20.0	592.3	681.2	589.0	45.9	30.1	-57.7
3b	23.3	54.6	43.3	1314.6	1496.1	1317.5	79.5	47.1	192.5
4b	2.4	-18.5	-4.5	1994.2	2482.4	1922.5	-127.2	290.9	93.1
5b	-258.1	32.2	-81.7	4642.5	3582.5	3716.3	-184.1	327.0	-31.2
6b	123.8	20.6	-4.6	2299.6	1890.3	1818.8	65.8	-190.7	66.0
7b	123.7	8.9	16.4	3001.0	2363.2	2328.5	-57.1	45.3	18.7
8b	-150.6	33.5	179.0	4596.2	4313.5	4393.3	-223.9	-167.6	184.3
9b	-86.4	45.5	2.6	3043.7	2569.7	2592.4	-315.6	-42.0	76.5
10b	-88.3	50.9	-79.5	2144.7	1872.7	2025.7	-223.5	322.1	-194.1
11b	-98.6	64.2	-80.4	2102.4	1874.6	1992.3	-310.8	306.1	-183.1
12b	-8.5	-13.5	15.6	1111.5	1256.8	1193.3	35.5	11.0	65.1
13b	-19.9	11.5	-16.7	523.3	547.1	577.0	-35.7	54.7	-74.7
GruPol									
1b	-76.6	3.1	-48.2	1734.7	1391.6	1543.0	-52.0	157.8	13.2
2b	14.2	35.0	-19.7	703.1	743.6	682.8	33.5	34.6	-50.7
3b	22.6	54.4	45.6	1520.5	1655.7	1572.7	19.7	11.7	90.0
4b	2.0	-14.9	-5.5	2408.4	2532.6	2296.1	-61.8	50.5	0.4
5b	-211.0	25.1	-70.8	4883.3	4240.9	4354.5	-125.4	205.8	-35.4
6b	106.5	10.9	-6.1	2340.8	2372.5	2448.9	-27.7	3.7	-6.9
7b	120.1	4.5	15.3	3137.0	2815.5	2817.1	-88.1	41.6	-4.2
8b	-137.6	16.9	148.0	5256.5	5231.6	5174.7	-82.9	-1.4	83.0
9b	-79.5	43.1	0.8	3189.0	3127.6	3010.4	-93.7	1.6	61.9
10b	-80.2	43.4	-69.2	2361.4	2201.2	2311.6	-101.3	119.9	-106.2
11b	-95.6	59.8	-73.4	2301.4	2178.7	2281.2	-182.0	147.4	-108.4
12b	-10.1	-12.0	15.8	1295.8	1457.7	1375.6	26.6	-10.2	45.6
13b	-18.2	9.7	-13.7	639.0	656.1	653.9	-3.8	18.9	14.0

S8 Building blocks entries and statistics

Table S8: Median (GruPol's entries, first line of each building block) and confidence interval (second line) at 95%, two tails, of the dipole moments and polarizabilites for all building blocks currently part of the GruPol Database. Count refers to the number of building blocks used to create a given entry.

Bulding Block	Count	μ_x	μ_y	μ_z	α_{xx}	α_{yy}	α_{zz}	α_{xy}	α_{xz}	α_{yz}
WATER	1	0.000	−0.747	0.000	9.342	8.452	7.892	0.000	0.000	0.000
		0.000	0.000	0.000	0.000	0.000	0.000	0.000	0.000	0.000
ALA_ZWI	16	−0.652	−1.599	0.188	24.709	22.830	18.563	−0.857	−0.615	1.001
		−0.109	−0.214	0.065	1.137	0.488	0.403	−1.121	−0.661	0.546
ARG_ZWI_1st	7	−0.451	−1.332	0.125	12.059	8.613	5.504	0.982	−0.392	0.238
		−0.160	−0.131	0.053	0.969	0.484	0.576	1.183	−0.173	0.516
ARG_ZWI_2nd	7	−0.040	−0.159	0.007	14.949	11.264	9.327	−0.891	−0.122	−1.230
		−0.196	−0.056	0.051	1.239	0.531	0.914	−0.390	−0.737	−0.415
ARG_ZWI_3rd	7	−0.129	−0.258	−0.005	17.518	10.792	9.960	−0.153	0.272	−0.186
		−0.165	−0.098	−0.111	1.051	1.300	1.608	−0.738	1.803	−1.141
ARG_ZWI_4th	7	−0.108	−0.006	0.114	10.579	9.103	13.087	−0.951	1.479	−0.371
		−0.063	−0.020	0.099	0.495	0.464	0.701	−0.323	0.437	−0.572
ARG_ZWI_5th	7	−0.209	0.029	−0.295	20.805	9.629	6.804	−1.410	2.061	0.403
		−0.120	0.134	−0.251	2.126	0.317	0.326	−1.158	1.344	0.264
ARG_ZWI_6th	7	−0.974	−0.802	−0.018	21.698	10.222	9.730	−0.519	−0.863	0.213
		−0.069	−0.055	−0.038	2.267	0.752	0.717	−1.278	−0.945	0.384
ARG_ZWI_7th	7	0.213	−0.029	−0.134	19.721	19.615	11.453	0.936	0.108	−0.409
		0.066	−0.115	−0.081	0.250	0.443	0.422	2.204	0.260	−0.697
ASN_ZWI_1st	7	−0.416	−1.325	0.212	10.571	8.018	5.167	1.962	−0.455	0.574
		−0.178	−0.144	0.038	0.475	0.529	0.425	0.735	−0.399	0.322
ASN_ZWI_2nd	7	−0.142	−0.060	−0.045	12.742	11.140	9.441	−0.047	0.673	−1.519
		−0.218	−0.046	−0.027	0.663	0.441	0.474	−0.306	0.371	−0.234
ASN_ZWI_3rd	7	0.080	−1.883	0.053	13.806	16.152	10.190	0.205	0.999	−2.217
		0.222	−0.032	0.038	0.625	1.676	1.122	0.728	0.513	−1.130
ASN_ZWI_4th	7	0.101	0.223	−0.043	15.315	10.796	9.353	3.988	1.160	0.403
		0.321	0.036	−0.054	1.023	0.757	0.651	0.876	1.021	0.744
ASP_ZWI_1st	7	−0.521	−1.455	0.200	10.308	8.443	5.124	2.741	−0.616	0.396
		−0.197	−0.227	0.032	0.477	0.650	0.460	1.226	−0.362	0.271
ASP_ZWI_2nd	7	−0.101	0.064	0.003	11.974	11.202	9.343	0.137	−0.029	−1.515
		−0.270	0.188	0.219	1.014	0.632	0.897	0.259	−0.719	−0.824

Bulding Block	Count	μ_x	μ_y	μ_z	α_{xx}	α_{yy}	α_{zz}	α_{xy}	α_{xz}	α_{yz}
CO_ACID_ZWI	13	−1.243	0.900	0.008	13.404	16.877	8.091	−4.698	−0.557	0.816
		−0.214	0.186	0.077	1.279	0.768	0.259	−0.535	−0.621	0.582
CTER_1	19	0.574	2.854	0.011	28.426	28.291	16.505	3.082	1.112	0.056
		0.143	0.145	0.127	1.309	1.000	0.907	0.731	1.113	0.326
CTER_2	9	−0.110	2.595	−0.153	28.065	28.403	15.902	−2.258	0.497	0.305
		−0.299	0.216	−0.238	2.677	0.944	1.520	−2.108	2.236	1.489
CYS_ZWI_1st	9	−0.536	−1.021	0.134	10.290	8.592	6.345	0.920	−0.460	0.111
		−0.081	−0.194	0.041	0.547	0.583	0.414	0.437	−0.351	0.289
CYS_ZWI_2nd	9	0.348	−0.122	−0.003	14.650	11.616	9.940	0.731	−0.133	−1.352
		0.174	−0.041	−0.037	1.128	0.348	0.525	0.588	−0.343	−0.198
CYS_ZWI_3rd	9	−0.701	−0.398	−0.013	23.590	20.083	20.311	1.871	2.954	2.055
		−0.189	−0.172	−0.093	2.200	1.137	1.051	2.483	2.162	1.037
GLN_ZWI_1st	7	−0.582	−1.329	0.131	11.767	8.509	6.359	1.616	−0.843	0.309
		−0.178	−0.189	0.045	1.267	1.149	0.465	1.154	−0.265	0.505
GLN_ZWI_2nd	7	−0.154	−0.084	0.008	14.295	11.227	9.375	−0.528	0.108	−1.099
		−0.234	−0.052	0.016	1.485	0.869	0.540	−0.358	0.682	−0.891
GLN_ZWI_3rd	7	−0.265	−0.254	0.024	14.296	11.221	9.230	−0.672	−0.308	0.014
		−0.233	−0.153	0.075	1.357	1.019	1.350	−1.263	−0.676	0.847
GLN_ZWI_4th	7	−0.200	−1.889	0.002	13.964	18.753	7.420	2.751	−0.328	−0.657
		−0.162	−0.085	0.006	1.505	1.780	0.409	0.395	−0.459	−0.691
GLN_ZWI_5th	7	−0.009	0.134	−0.003	15.309	13.009	8.666	3.705	0.012	0.106
		−0.142	0.047	−0.026	0.616	1.002	0.367	1.550	0.350	0.801
GLU_ZWI_1st	6	−0.436	−1.581	0.139	11.198	9.457	5.947	0.153	−0.816	0.361
		−0.151	−0.394	0.032	1.062	1.089	0.999	1.747	−0.537	0.435
GLU_ZWI_2nd	6	−0.094	−0.029	−0.064	13.610	11.786	9.303	−0.384	0.551	−0.320
		−0.208	−0.127	−0.101	0.904	0.514	0.425	−0.575	0.715	−1.730
GLU_ZWI_3rd	6	−0.112	−0.167	−0.051	14.234	12.065	8.700	−0.341	0.193	0.848
		−0.190	−0.160	−0.084	0.776	0.921	0.510	−0.452	0.517	1.172
GLY_ZWI	20	−1.661	0.126	−0.032	11.205	9.955	8.088	1.096	−0.273	0.297
		−0.199	0.051	−0.044	0.944	0.478	0.286	0.495	−0.355	0.331
HEME_CH_1	4	0.022	−0.126	−0.005	31.973	13.965	5.786	0.956	−0.453	0.012
		0.028	−0.008	−0.015	1.123	1.569	0.184	1.207	−1.200	0.446
HEME_FE	1	−0.050	0.060	−0.349	25.096	22.295	5.253	−0.509	−1.118	0.872
		0.000	0.000	0.000	0.000	0.000	0.000	0.000	0.000	0.000
HEME_N_RING_1	2	−0.084	−0.093	−0.125	86.931	102.216	19.862	−2.840	3.833	−2.507

**Bulding Block**	**Count**	μ_x	μ_y	μ_z	α_{xx}	α_{yy}	α_{zz}	α_{xy}	α_{xz}	α_{yz}
		0.000	0.000	0.000	0.000	0.000	0.000	0.000	0.000	0.000
HEME_N_RING_2	2	−0.061	−0.131	−0.064	99.749	100.506	18.988	−1.103	2.216	0.075
		0.000	0.000	0.000	0.000	0.000	0.000	0.000	0.000	0.000
HEME_SUBS_C2H3_1	2	0.111	−0.101	−0.001	59.165	22.318	18.755	0.076	3.533	−1.231
		0.000	0.000	0.000	0.000	0.000	0.000	0.000	0.000	0.000
HEME_SUBS_CH2_1	2	0.002	−0.132	0.005	17.475	13.993	11.237	−3.496	−2.746	1.992
		0.000	0.000	0.000	0.000	0.000	0.000	0.000	0.000	0.000
HEME_SUBS_CH2_2	2	0.166	−0.077	−0.073	14.999	12.008	9.299	−0.732	−0.135	1.899
		0.000	0.000	0.000	0.000	0.000	0.000	0.000	0.000	0.000
HEME_SUBS_CH3_1	4	−0.117	−0.011	−0.016	22.002	12.138	11.570	−2.026	0.577	0.507
		−0.036	−0.032	−0.035	0.799	3.763	0.637	−3.773	2.523	0.705
HEME_SUBS_COO	2	−0.891	0.402	0.019	20.273	26.638	14.874	−1.839	0.881	−1.723
		0.000	0.000	0.000	0.000	0.000	0.000	0.000	0.000	0.000
HSD_ZWI_1st	5	−0.482	−1.474	0.129	10.446	9.702	5.819	0.189	−0.324	0.609
		−0.308	−0.333	0.038	1.327	0.729	0.876	1.455	−0.569	0.722
HSD_ZWI_2nd	5	−0.009	−0.022	−0.045	13.560	12.345	8.968	−1.321	−0.203	0.934
		−0.247	−0.037	−0.027	0.712	0.898	0.688	−0.763	−0.500	1.102
ILE_ZWI_1st	7	−0.422	−1.506	0.153	10.164	8.699	6.676	0.575	−1.298	1.059
		−0.103	−0.148	0.024	1.846	0.640	0.684	0.780	−0.279	0.438
ILE_ZWI_2nd	7	0.017	0.142	0.029	10.282	10.172	9.984	0.119	−0.259	−0.483
		0.056	0.092	0.051	0.935	0.470	0.553	0.846	−0.710	−1.003
ILE_ZWI_3rd	7	−0.107	−0.049	−0.129	14.315	11.410	13.316	−0.120	1.173	−0.053
		−0.088	−0.027	−0.047	0.863	0.268	0.345	−0.163	0.503	−0.364
ILE_ZWI_4th	7	0.119	−0.407	−0.033	14.575	13.416	9.654	−1.021	−0.679	1.254
		0.071	−0.097	−0.028	0.705	0.901	0.458	−0.405	−0.399	0.413
ILE_ZWI_5th	7	−0.135	−0.114	−0.075	17.839	12.267	13.106	1.057	1.307	0.935
		−0.064	−0.017	−0.009	1.158	0.130	0.313	0.088	0.275	0.220
IMI_ZWI	5	1.205	−1.261	−0.099	58.380	45.393	31.636	−0.916	0.495	2.848
		0.633	−0.324	−0.311	1.374	7.483	1.376	−1.772	1.684	3.738
LEU_ZWI_1st	8	−0.503	−1.371	0.147	11.715	8.118	5.017	1.897	−0.219	0.588
		−0.056	−0.165	0.030	0.788	0.459	0.477	0.494	−0.242	0.236
LEU_ZWI_2nd	8	−0.114	−0.134	0.017	13.914	12.170	9.675	−0.896	0.297	−1.436
		−0.074	−0.019	0.059	0.271	0.253	0.253	−0.434	0.495	−0.171
LEU_ZWI_3rd	8	−0.166	0.095	0.016	11.633	10.544	8.699	−0.417	−0.486	−0.122
		−0.053	0.020	0.039	0.369	0.326	0.202	−0.176	−0.486	−0.241

Bulding Block	Count	μ_x	μ_y	μ_z	α_{xx}	α_{yy}	α_{zz}	α_{xy}	α_{xz}	α_{yz}
LEU_ZWI_4th	8	−0.173	−0.252	−0.020	15.351	13.344	15.001	−1.330	1.482	−1.095
		−0.045	−0.059	−0.042	0.828	0.226	0.745	−0.175	0.249	−0.631
LEU_ZWI_5th	8	−0.173	−0.249	0.020	16.716	13.949	13.469	−0.363	1.608	−1.718
		−0.080	−0.034	0.038	0.692	0.355	0.257	−0.595	0.513	−0.493
LYS_ZWI_1st	7	−0.459	−1.269	0.143	13.145	8.959	5.784	1.692	−0.587	0.290
		−0.283	−0.189	0.023	1.291	1.186	0.611	1.477	−0.310	0.476
LYS_ZWI_2nd	7	−0.164	−0.099	0.074	15.797	11.738	9.436	−0.402	−0.177	−0.791
		−0.297	−0.131	0.092	1.623	0.489	0.424	−0.707	−0.991	−1.170
LYS_ZWI_3rd	7	−0.086	−0.182	0.025	16.513	10.511	9.490	−0.195	−0.072	−0.763
		−0.292	−0.089	0.043	1.554	0.842	0.661	−0.934	−0.600	−0.810
LYS_ZWI_4th	7	−0.099	−0.240	0.016	16.523	11.302	10.211	−0.134	−0.220	0.052
		−0.257	−0.058	0.078	1.595	1.057	1.074	−0.438	−0.477	0.987
LYS_ZWI_5th	7	−0.094	−0.053	0.241	9.505	9.892	14.295	0.325	0.916	0.031
		−0.104	−0.064	0.183	0.511	0.470	0.523	0.717	1.222	0.728
LYS_ZWI_6th	7	−0.493	0.009	−0.652	19.071	10.861	11.345	−0.637	−0.477	0.561
		−0.182	0.137	−0.096	0.408	1.073	2.318	−1.611	−1.820	0.882
MET_ZWI_1st	8	−0.520	−1.505	0.172	11.966	8.731	5.590	1.680	−0.672	0.665
		−0.156	−0.135	0.041	0.703	0.989	0.569	0.965	−0.208	0.470
MET_ZWI_2nd	8	−0.022	−0.089	−0.001	14.586	11.355	9.061	−0.924	0.153	−1.229
		−0.123	−0.106	−0.046	0.841	0.806	0.903	−0.327	0.378	−0.634
MET_ZWI_3rd	8	−0.053	−0.283	0.044	20.729	11.462	8.828	1.099	−0.325	−0.425
		−0.114	−0.073	0.085	0.779	0.894	1.246	0.977	−1.209	−1.013
MET_ZWI_4th	8	−0.199	−0.766	−0.003	23.210	21.451	16.026	−1.601	−0.129	0.205
		−0.117	−0.138	−0.051	1.332	1.303	0.459	−0.761	−0.834	0.932
MET_ZWI_5th	8	−0.035	−0.019	−0.002	20.335	13.457	11.848	1.930	0.359	−0.452
		−0.091	−0.121	−0.063	1.466	0.988	0.442	0.774	0.500	−0.839
NTER_1	24	1.427	0.368	−0.051	13.122	8.896	7.892	2.036	1.411	0.919
		0.077	0.065	−0.029	0.517	0.252	0.089	0.318	0.249	0.182
NTER_2	4	1.266	0.317	−0.119	14.366	7.846	8.471	0.046	−0.529	−0.496
		0.212	0.241	−0.206	2.465	0.323	1.684	0.661	−0.796	−0.205
OH_ZWI_ACID	13	−0.408	0.830	−0.016	11.633	6.455	5.524	1.186	−0.080	−0.114
		−0.096	0.070	−0.037	0.837	0.434	0.797	0.420	−0.343	−0.367
OH_ZWI_PHE	5	−0.933	0.403	0.013	17.120	7.097	5.601	−0.057	0.036	−0.061
		−0.289	0.257	0.052	1.605	2.657	0.672	−1.576	0.587	−0.829
OH_ZWI_SER	8	−0.758	0.671	0.007	13.332	8.358	5.804	0.354	0.351	−0.143

**Bulding Block**	**Count**	μ_x	μ_y	μ_z	α_{xx}	α_{yy}	α_{zz}	α_{xy}	α_{xz}	α_{yz}
		−0.248	0.166	0.034	0.817	0.906	0.433	1.268	0.602	−0.757
OH_ZWI_THR	6	−0.505	0.786	0.042	12.514	7.949	5.085	0.738	0.001	0.107
		−0.268	0.224	0.109	1.105	1.474	0.679	1.023	1.374	0.725
PEPT_BOND_CO_ZWI_0	27	−1.125	2.321	0.066	15.597	18.568	7.052	−1.123	0.027	0.459
		−0.152	0.172	0.042	0.708	1.143	0.378	−0.926	0.278	0.636
PEPT_BOND_CO_ZWI_1	86	−0.798	2.158	0.048	15.723	17.689	6.556	1.594	0.691	0.034
		−0.066	0.043	0.021	0.298	0.406	0.210	0.297	0.271	0.187
PEPT_BOND_CO_ZWI_2	9	−1.280	2.083	0.023	15.553	19.701	7.185	0.107	0.161	−0.337
		−0.377	0.274	0.118	1.280	1.292	0.699	2.003	1.262	−1.288
PEPT_BOND_CO_ZWI_3	7	−2.056	1.997	0.160	18.646	16.427	7.919	−5.734	0.526	0.183
		−0.536	0.550	0.221	1.256	2.956	0.467	−1.362	1.410	0.604
PEPT_BOND_NH_ZWI_0	18	−1.155	1.841	0.042	13.973	14.334	6.167	−6.041	−0.212	−0.198
		−0.157	0.212	0.031	0.732	0.926	0.270	−1.043	−0.557	−0.666
PEPT_BOND_NH_ZWI_1	65	−1.025	1.714	0.035	14.392	17.301	5.865	−7.707	0.625	0.040
		−0.074	0.070	0.028	0.368	0.412	0.210	−0.356	0.358	0.313
PEPT_BOND_NH_ZWI_2	33	−1.268	1.519	0.011	15.368	17.959	5.827	−8.088	1.021	0.410
		−0.115	0.142	0.031	0.473	0.744	0.246	−0.651	0.409	0.322
PEPT_BOND_NH_ZWI_3	6	−0.647	1.580	0.060	13.761	15.432	5.694	−3.579	0.375	0.865
		−0.446	0.534	0.121	1.712	2.302	0.762	−1.993	0.725	1.727
PEPT_BOND_NH_ZWI_4	7	0.295	0.879	0.086	14.180	12.007	5.585	−1.172	0.423	0.770
		0.212	0.212	0.061	0.572	0.606	0.282	−0.855	0.744	0.582
PHENOLIC_ZWI	5	−1.036	−0.049	−0.199	80.553	66.354	38.936	−1.296	7.003	0.044
		−0.579	−0.078	−0.326	7.976	2.207	4.478	−2.465	3.799	1.937
PHE_ZWI_1st	8	−0.385	−1.266	0.155	13.602	8.139	5.548	1.504	0.229	0.252
		−0.112	−0.178	0.021	1.069	0.785	0.229	0.328	0.182	0.308
PHE_ZWI_2nd	8	−0.119	−0.152	0.035	16.818	12.148	8.789	−1.885	0.579	−0.915
		−0.041	−0.034	0.079	1.351	0.271	0.684	−0.330	0.541	−0.322
PHE_ZWI_3rd	8	0.549	−0.163	−0.071	97.136	72.148	40.025	0.056	−4.638	−0.745
		0.174	−0.182	−0.118	3.282	1.751	1.641	2.058	−1.764	−1.414
PRO_ZWI_1st	5	−2.278	2.671	−0.345	39.611	26.268	11.008	−0.598	0.281	1.424
		−0.521	0.316	−0.357	4.468	0.836	1.007	−2.224	1.788	1.022
PRO_ZWI_2nd	5	−1.400	0.325	1.050	41.132	45.884	38.505	−1.661	−3.143	−3.888
		−0.293	0.125	0.189	2.690	2.779	1.149	−1.450	−1.269	−1.363
SER_ZWI_1st	8	−0.562	−0.908	0.089	10.465	7.790	6.102	1.138	−0.339	0.395
		−0.187	−0.238	0.061	0.604	0.465	0.466	0.996	−0.182	0.210

Bulding Block	Count	μ_x	μ_y	μ_z	α_{xx}	α_{yy}	α_{zz}	α_{xy}	α_{xz}	α_{yz}
SER_ZWI_2nd	8	0.108	−0.192	−0.026	9.718	10.532	9.241	−2.153	−0.201	−0.767
		0.178	−0.240	−0.093	0.548	0.466	0.293	−0.507	−0.623	−0.504
THR_ZWI_1st	6	−0.461	−1.266	0.124	9.876	8.092	5.754	0.342	−0.060	0.234
		−0.109	−0.348	0.017	0.759	1.244	0.851	0.266	−0.499	0.614
THR_ZWI_2nd	6	−0.181	−0.060	0.067	6.939	8.005	9.222	−1.148	−0.628	1.010
		−0.172	−0.186	0.074	0.372	0.456	1.014	−0.532	−0.523	0.541
THR_ZWI_3rd	6	−0.116	−0.026	−0.206	16.111	12.240	14.081	−0.845	0.225	0.324
		−0.078	−0.086	−0.116	1.509	0.600	1.022	−0.615	1.071	1.091
TRP_ZWI_1st	10	−0.510	−1.182	0.152	11.899	7.671	5.193	1.545	0.713	0.529
		−0.148	−0.113	0.023	0.875	0.907	0.507	0.732	0.262	0.337
TRP_ZWI_2nd	10	−0.159	−0.100	−0.009	13.589	13.031	9.178	−1.972	−0.612	−0.916
		−0.090	−0.020	−0.051	0.940	0.936	0.590	−0.338	−0.360	−0.302
TRP_ZWI_3rd	10	−0.143	−0.090	0.043	23.457	23.834	14.302	−0.283	−1.204	−0.193
		−0.117	−0.081	0.102	0.947	1.109	1.182	−0.638	−0.957	−0.454
TRP_ZWI_4th	10	−0.076	−0.230	−0.087	16.395	13.538	7.691	1.083	−1.414	0.497
		−0.050	−0.157	−0.103	0.564	0.788	0.675	0.341	−0.752	0.579
TRP_ZWI_5th	10	0.794	−0.077	0.016	81.297	79.923	36.903	−10.380	−5.382	3.894
		0.066	−0.236	0.055	1.697	3.557	0.384	−1.643	−1.432	0.515
TYR_ZWI_1st	5	−0.722	−1.088	0.166	11.820	9.035	5.631	1.998	−0.180	0.498
		−0.228	−0.357	0.031	1.923	1.812	1.377	1.238	−0.317	0.780
TYR_ZWI_2nd	5	−0.326	−0.060	0.036	14.940	13.087	8.699	−1.402	0.099	−0.959
		−0.240	−0.136	0.094	2.382	0.855	0.784	−0.937	0.582	−0.452
VAL_ZWI_1st	6	−0.413	−1.402	0.110	10.476	8.478	5.891	1.346	−0.987	0.783
		−0.097	−0.114	0.032	0.932	0.553	0.639	0.674	−0.181	0.303
VAL_ZWI_2nd	6	−0.083	−0.039	−0.091	9.043	10.382	9.675	−0.044	−0.728	0.054
		−0.051	−0.096	−0.143	0.475	0.537	0.909	−0.224	−0.278	0.888
VAL_ZWI_3rd	6	−0.104	−0.282	−0.011	16.813	14.727	12.127	−0.250	1.352	−0.922
		−0.073	−0.053	−0.020	1.121	0.419	0.465	−0.300	0.211	−0.496
VAL_ZWI_4th	6	−0.134	−0.033	−0.089	15.772	11.660	13.107	−0.494	0.464	−0.812
		−0.077	−0.037	−0.042	0.691	0.285	0.281	−0.287	0.503	−0.587

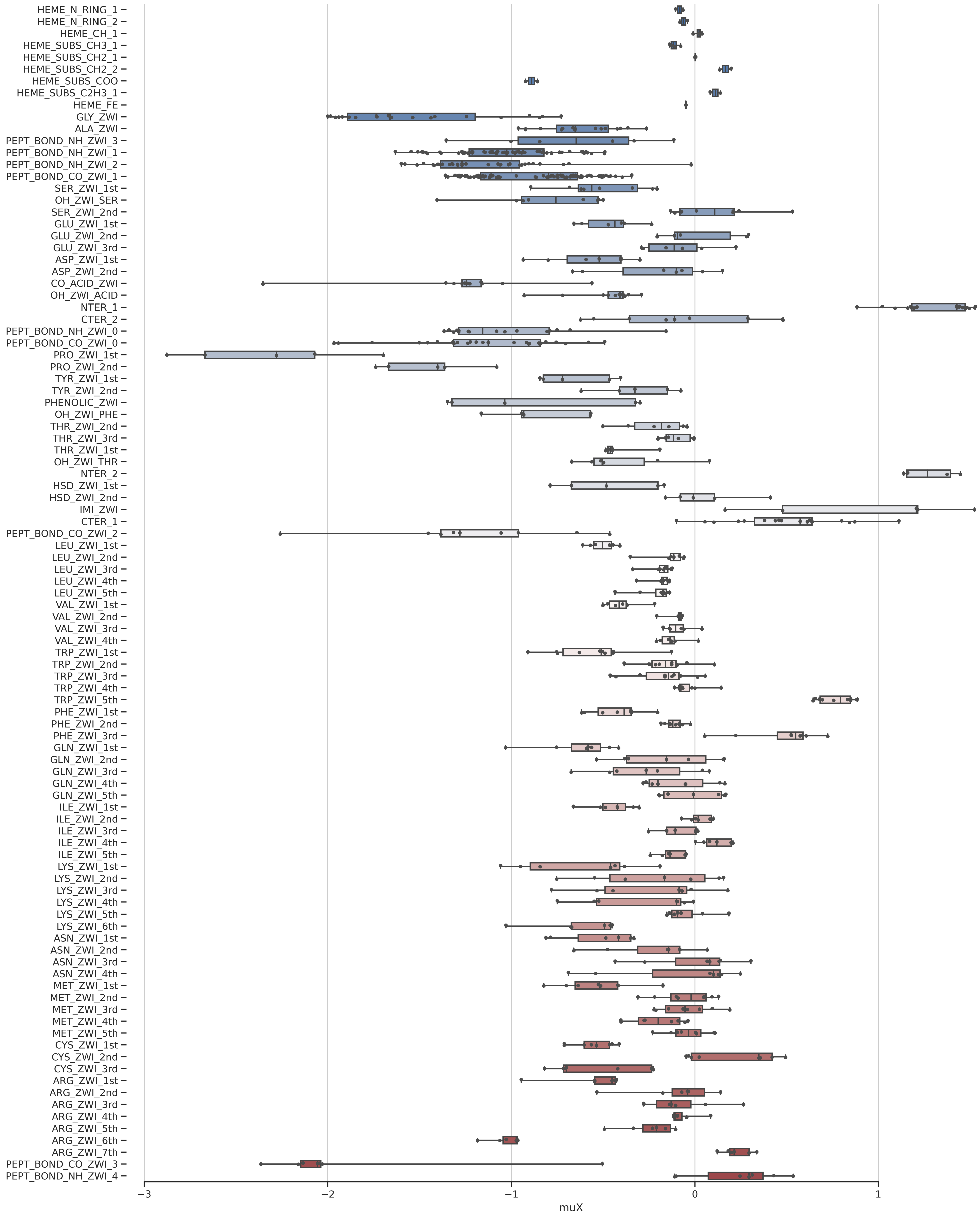

Figure S8: The boxplot visually represents the quartiles of the x component of the dipole moment for each building block stored within GruPol, with whiskers extending to display the remainder of the distribution, based on a method tied to the inter-quartile range (see M. L. Waskom, 2021)

[3]M. L. Waskom, JOSS, **2021**, 6(60), 3021

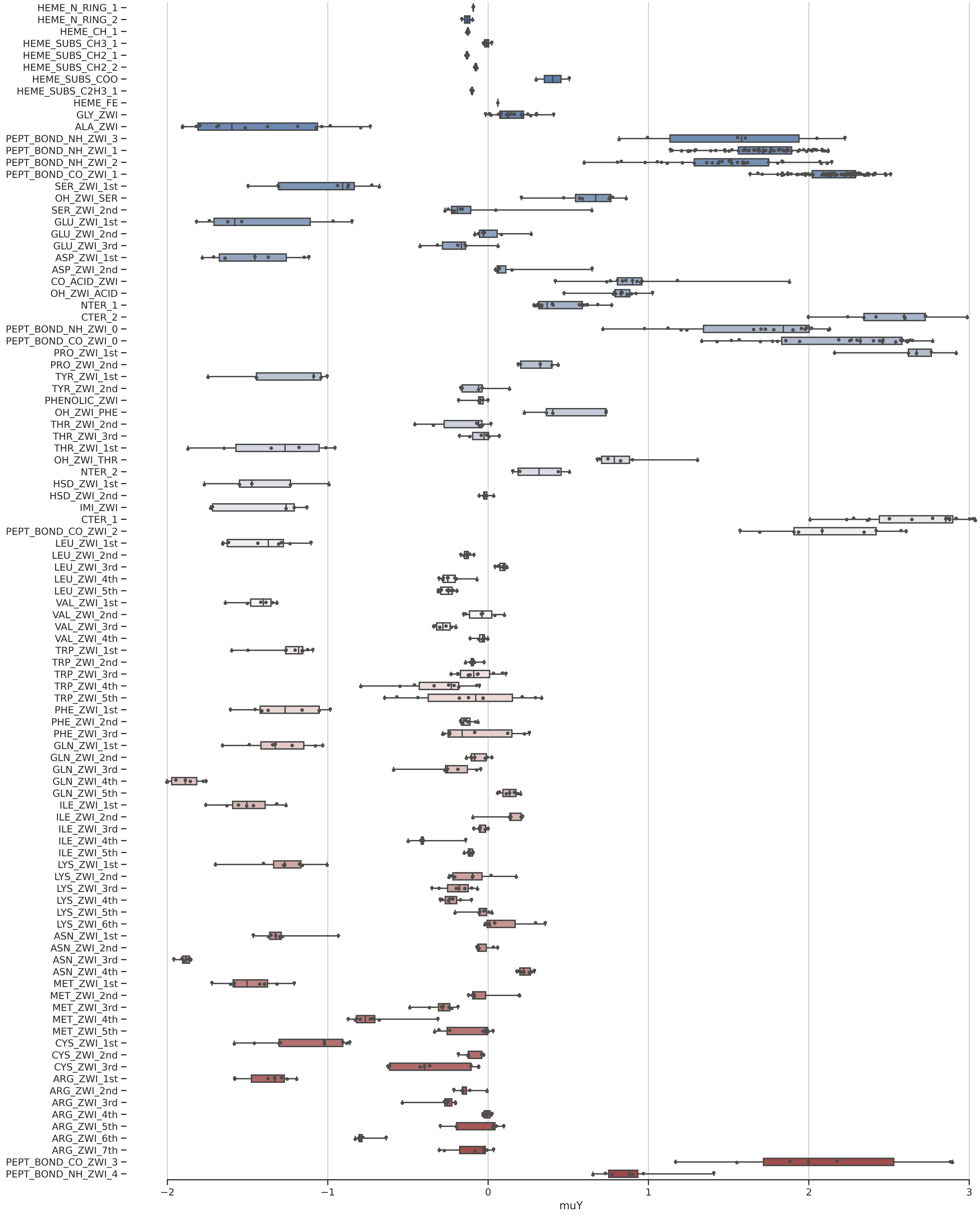

Figure S9: The boxplot visually represents the quartiles of the y component of the dipole moment for each building block stored within GruPol, with whiskers extending to display the remainder of the distribution, based on a method tied to the inter-quartile range (see M. L. Waskom, 2021)

[4]M. L. Waskom, JOSS, **2021**, 6(60), 3021

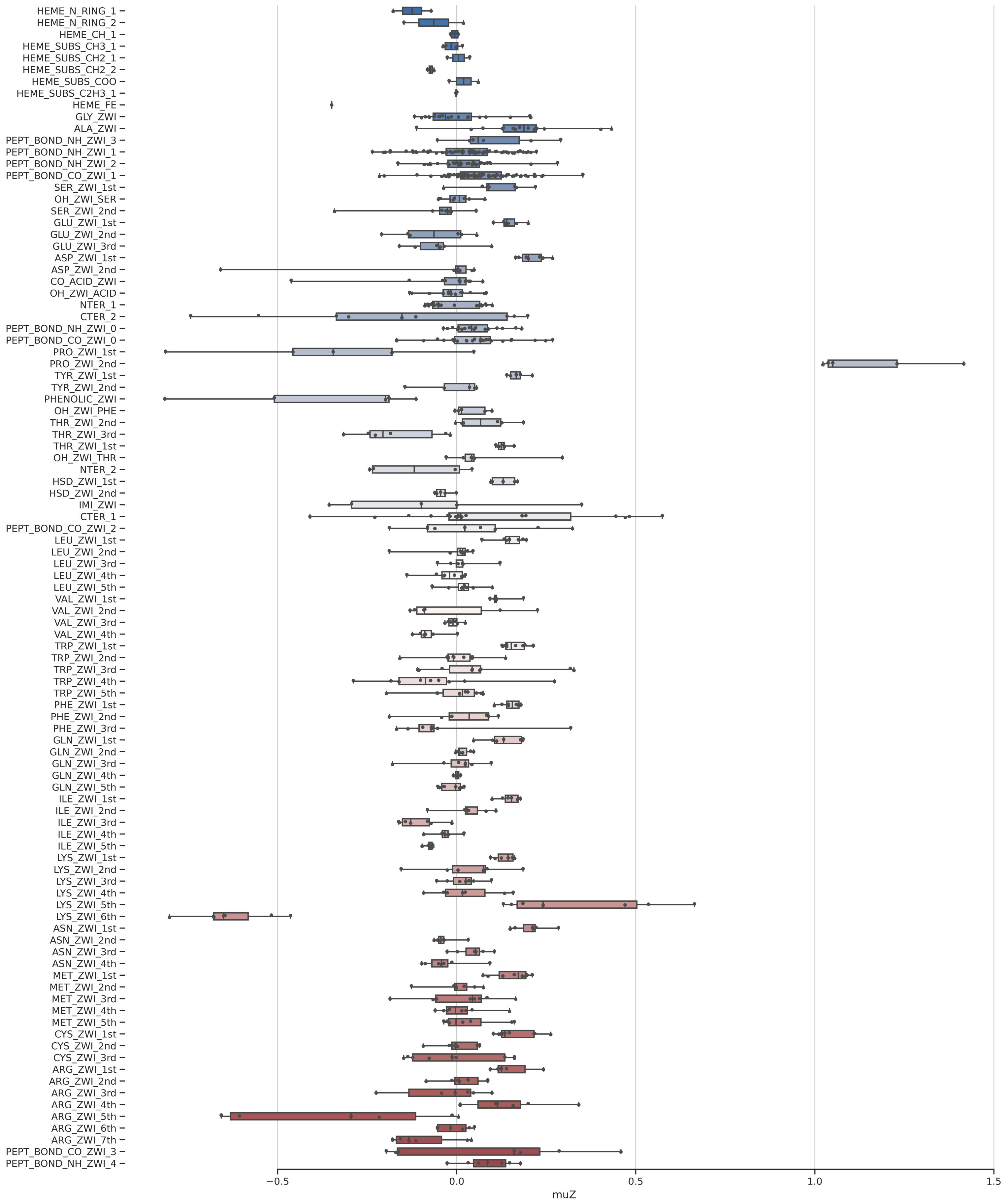

Figure S10: The boxplot visually represents the quartiles of the z component of the dipole moment for each building block stored within GruPol, with whiskers extending to display the remainder of the distribution, based on a method tied to the inter-quartile range (see M. L. Waskom, 2021)

[5]M. L. Waskom, JOSS, **2021**, 6(60), 3021

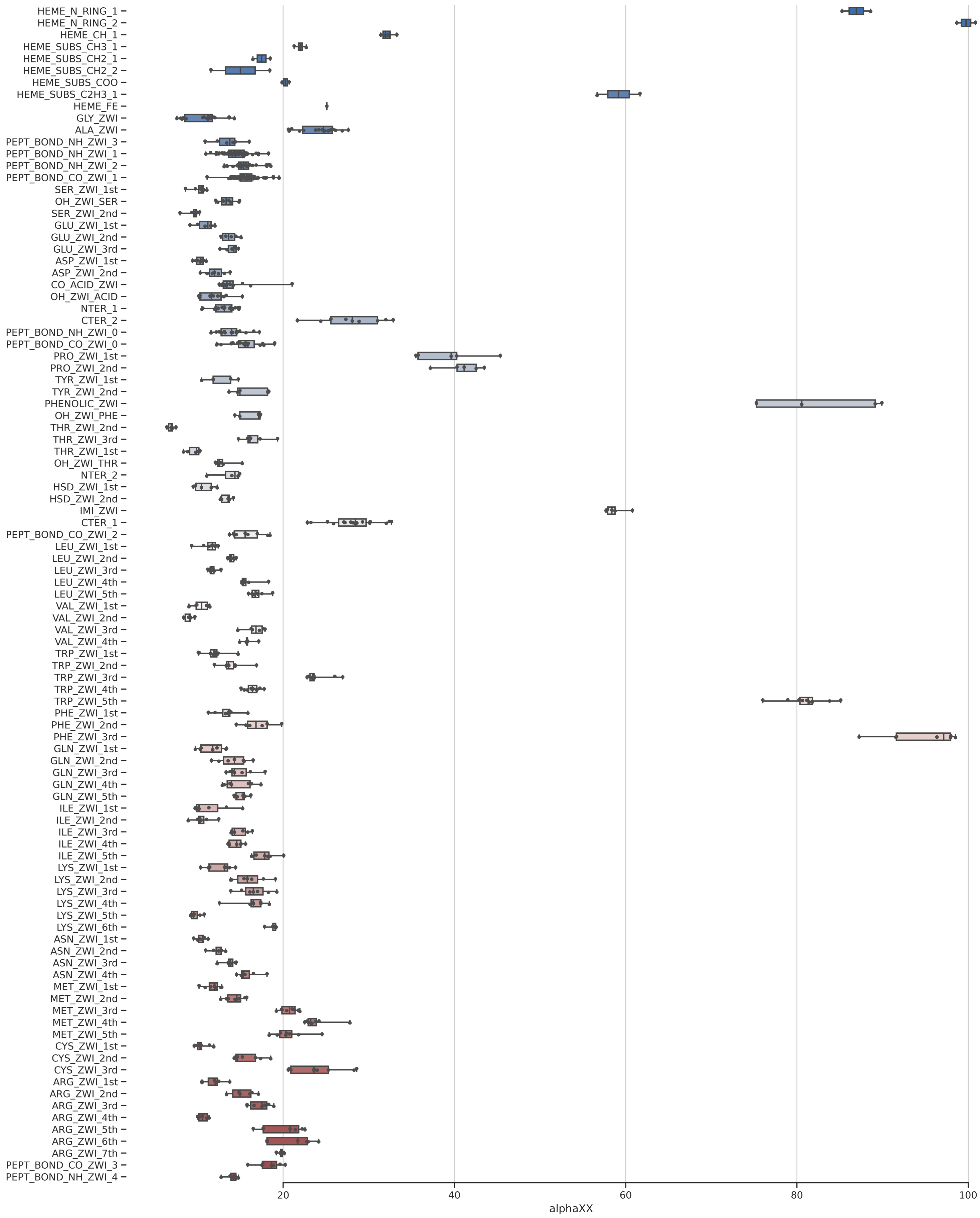

Figure S11: The boxplot visually represents the quartiles of the xx component of the polarizability for each building block stored within GruPol, with whiskers extending to display the remainder of the distribution, based on a method tied to the inter-quartile range (see M. L. Waskom, 2021)

[6] M. L. Waskom, JOSS, **2021**, 6(60), 3021

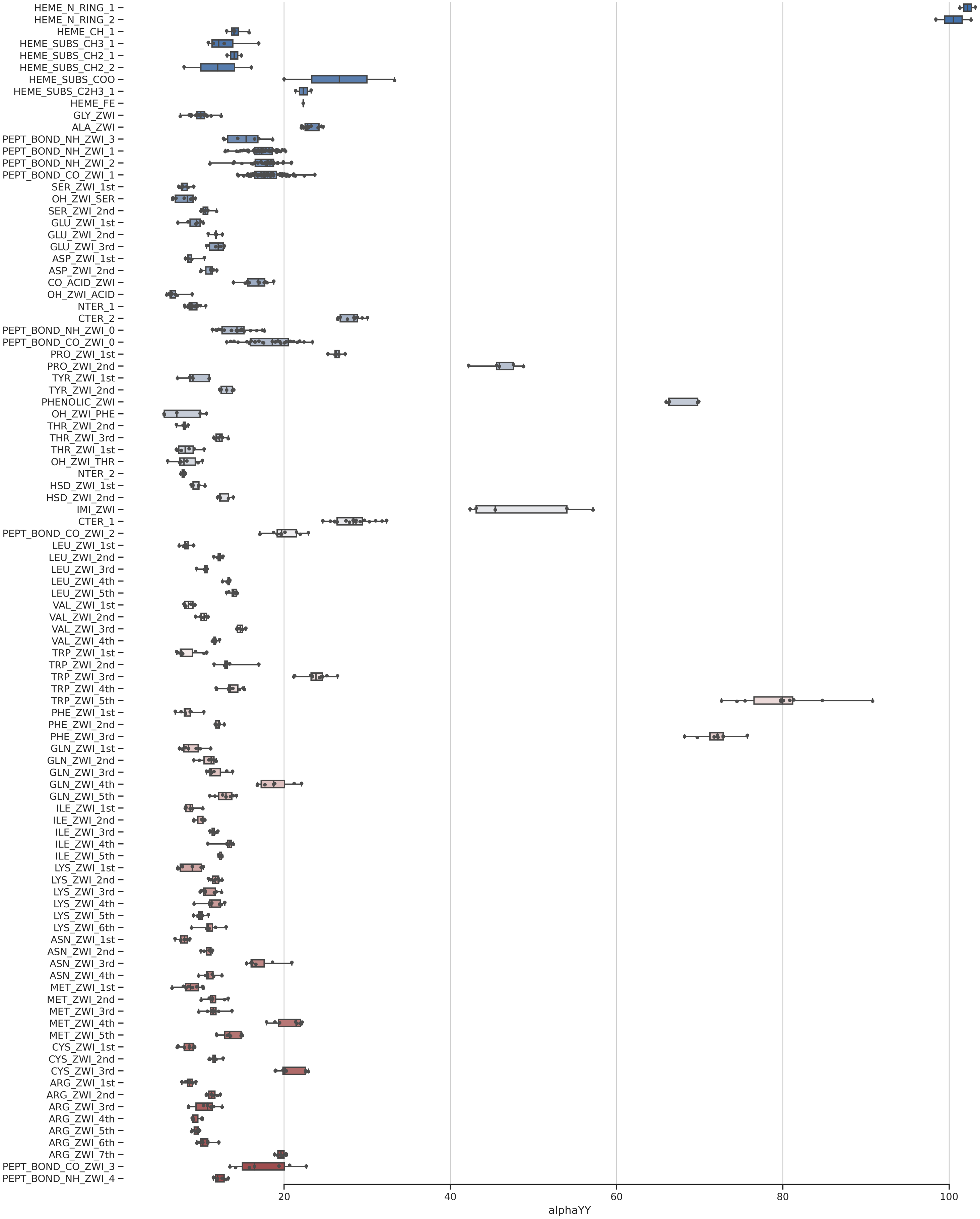

Figure S12: The boxplot visually represents the quartiles of the yy component of the polarizability for each building block stored within GruPol, with whiskers extending to display the remainder of the distribution, based on a method tied to the inter-quartile range (see M. L. Waskom, 2021)

[7]M. L. Waskom, JOSS, **2021**, 6(60), 3021

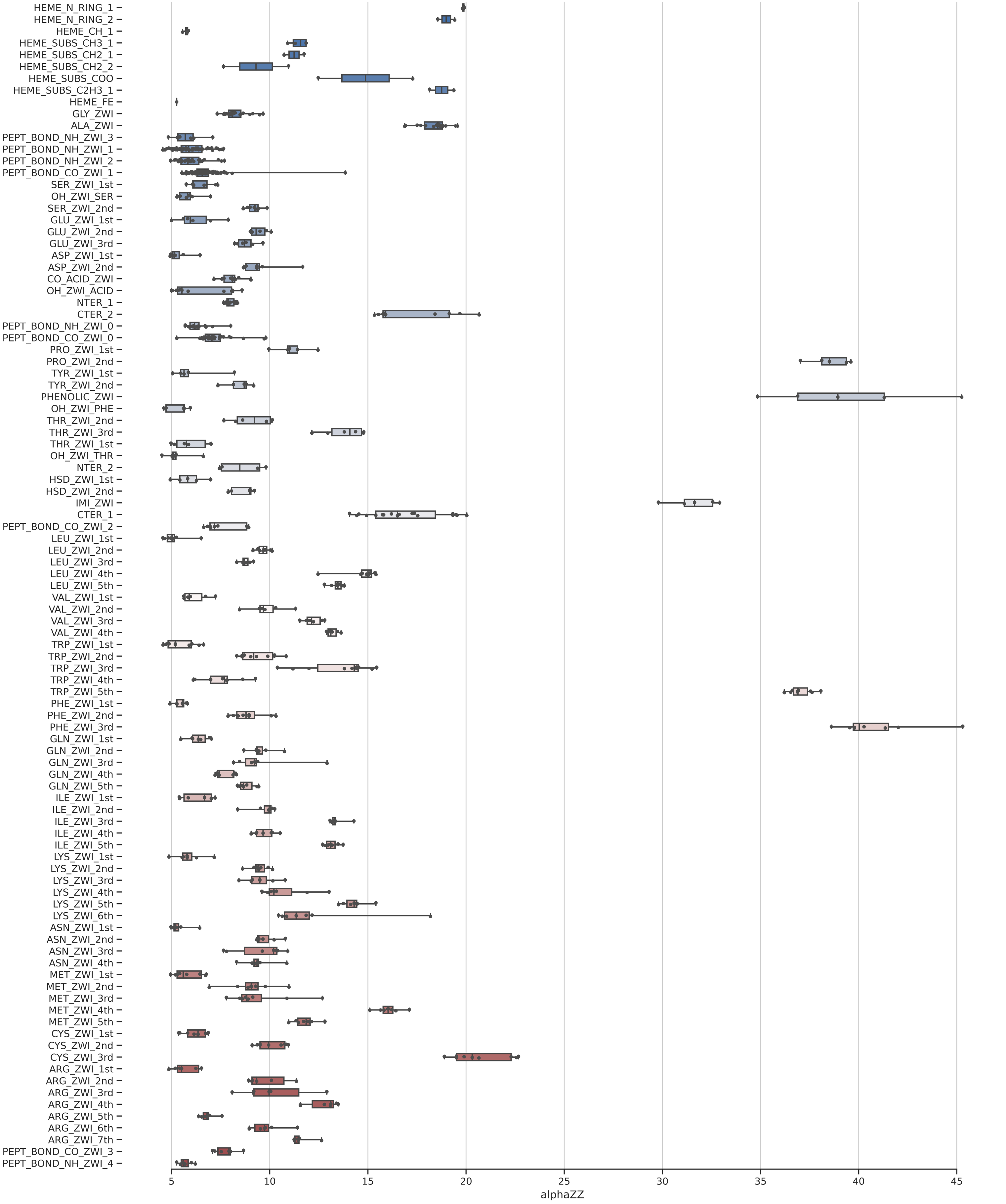

Figure S13: The boxplot visually represents the quartiles of the zz component of the polarizability for each building block stored within GruPol, with whiskers extending to display the remainder of the distribution, based on a method tied to the inter-quartile range (see M. L. Waskom, 2021)

[8]M. L. Waskom, JOSS, **2021**, 6(60), 3021

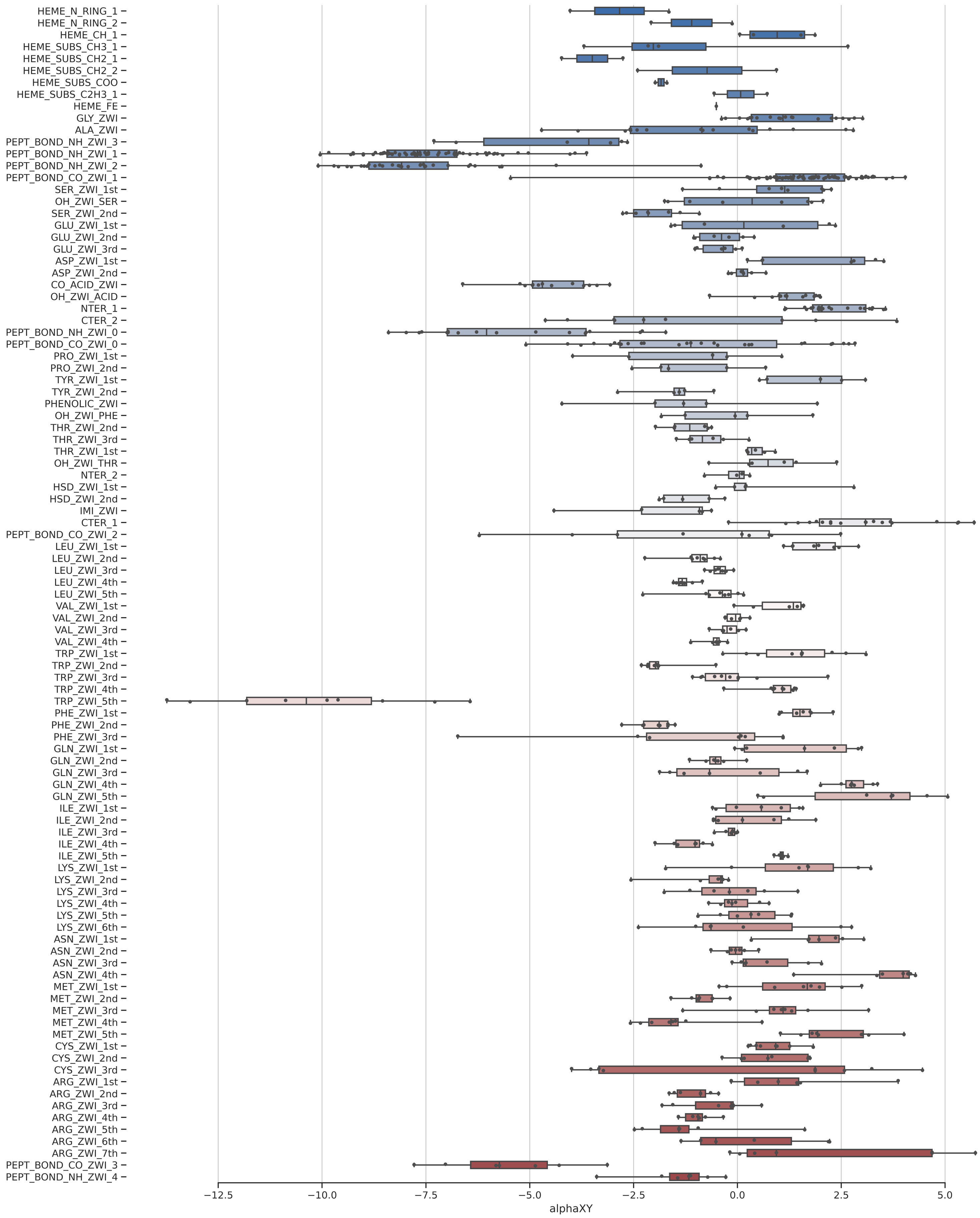

Figure S14: The boxplot visually represents the quartiles of the xy component of the polarizability for each building block stored within GruPol, with whiskers extending to display the remainder of the distribution, based on a method tied to the inter-quartile range (see M. L. Waskom, 2021)

[9] M. L. Waskom, JOSS, **2021**, 6(60), 3021

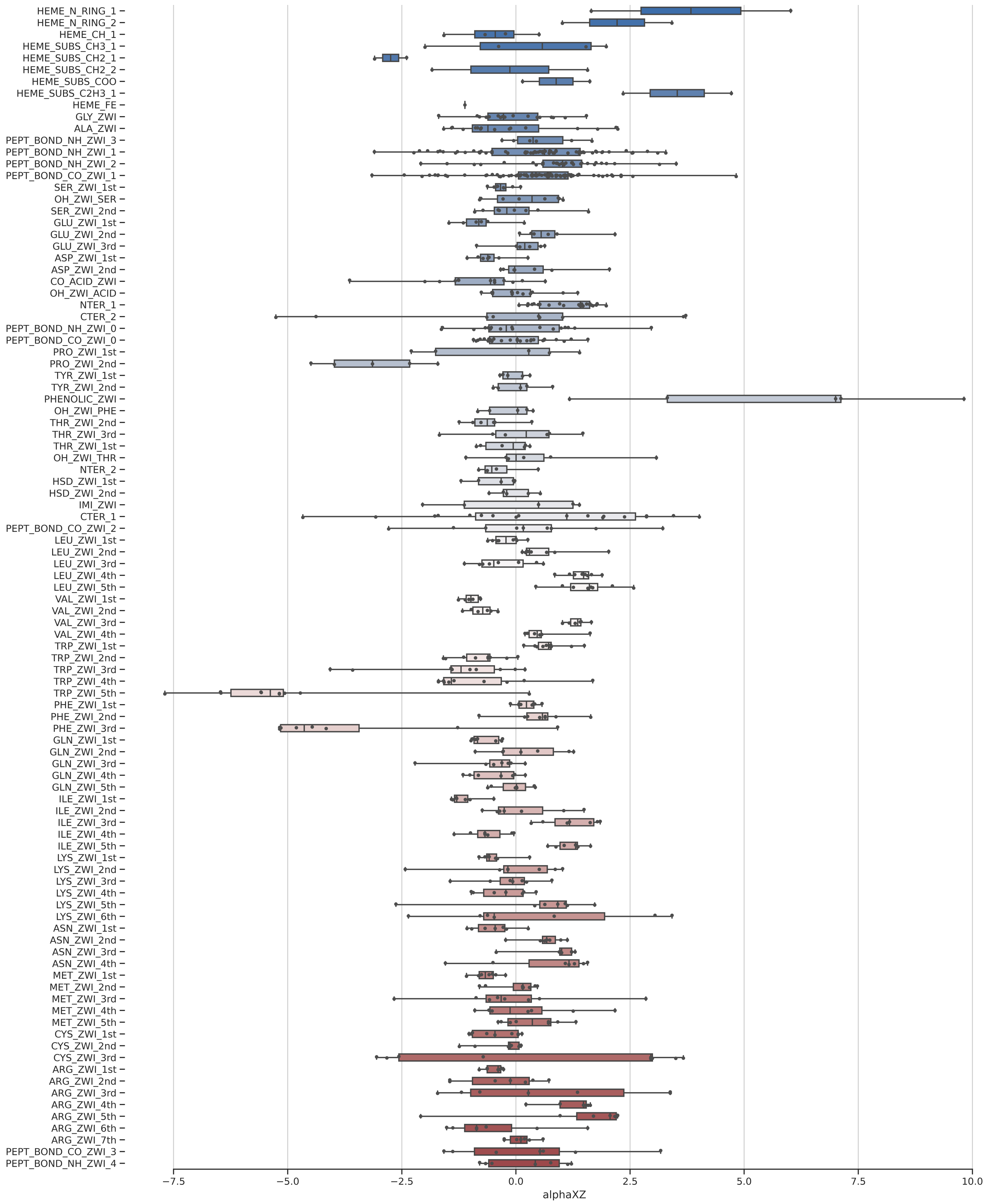

Figure S15: The boxplot visually represents the quartiles of the xz component of the polarizability for each building block stored within GruPol, with whiskers extending to display the remainder of the distribution, based on a method tied to the inter-quartile range (see M. L. Waskom, 2021)

[10]M. L. Waskom, JOSS, **2021**, 6(60), 3021

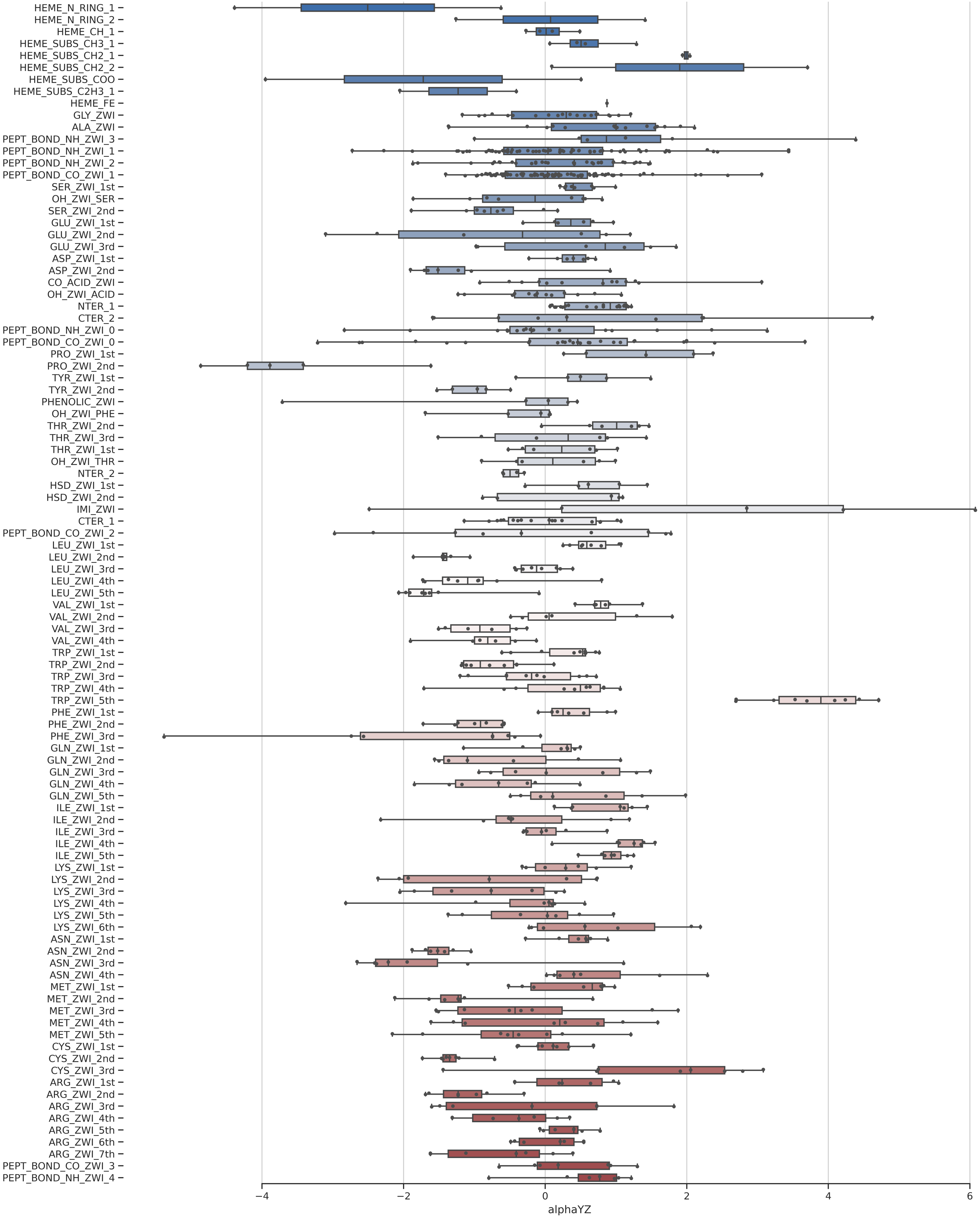

Figure S16: The boxplot visually represents the quartiles of the yz component of the polarizability for each building block stored within GruPol, with whiskers extending to display the remainder of the distribution, based on a method tied to the inter-quartile range (see M. L. Waskom, 2021)

[11]M. L. Waskom, JOSS, **2021**, 6(60), 3021
